# Paleomagnetic evidence for a long-lived, potentially reversing martian dynamo at ~3.9 Ga

**DOI:** 10.1126/sciadv.ade9071

**Published:** 2023-05-24

**Authors:** Sarah C. Steele, Roger R. Fu, Michael W. R. Volk, Thomas L. North, Alec R. Brenner, Adrian R. Muxworthy, Gareth S. Collins, Thomas M. Davison

**Affiliations:** ^1^ Department of Earth and Planetary Sciences, Harvard University, Cambridge, MA 02138, USA.; ^2^ Department of Earth Sciences, Utrecht University, Utrecht 3584 CS, Netherlands.; ^3^ Department of Earth Science and Engineering, Imperial College London, London SW7 2AZ, UK.

## Abstract

The 4.1-billion-year-old meteorite Allan Hills 84001 (ALH 84001) may preserve a magnetic record of the extinct martian dynamo. However, previous paleomagnetic studies have reported heterogeneous, nonunidirectional magnetization in the meteorite at submillimeter scales, calling into question whether it records a dynamo field. We use the quantum diamond microscope to analyze igneous Fe-sulfides in ALH 84001 that may carry remanence as old as 4.1 billion years (Ga). We find that individual, 100-μm-scale ferromagnetic mineral assemblages are strongly magnetized in two nearly antipodal directions. This suggests that the meteorite recorded strong fields following impact heating at 4.1 to 3.95 Ga, after which at least one further impact heterogeneously remagnetized the meteorite in a nearly antipodal local field. These observations are most simply explained by a reversing martian dynamo that was active until 3.9 Ga, thereby implying a late cessation for the martian dynamo and potentially documenting reversing behavior in a nonterrestrial planetary dynamo.

## Teaser

The paleomagnetic record in the ancient martian meteorite ALH 84001 suggests Mars may have had a long-lived, reversing dynamo.

## INTRODUCTION

Although Mars does not currently host a core dynamo, intense crustal magnetic fields associated with many of its ancient terrains indicate that a dynamo was active early in its past (*1*). However, extracting concrete information about that dynamo’s history from orbital measurements of crustal fields has been complicated by the high altitude and low spatial resolution of available measurements, imprecise dating of surface features, and poorly defined magnetic source regions. Several important properties of the dynamo therefore remain uncertain.

A central remaining question is the timing of the dynamo’s cessation. Weak magnetic fields above large ancient impact basins such as Hellas, Argyre, and Isidis have been widely interpreted as evidence that the martian dynamo shut down 4.1 to 4.0 billion years (Ga) ago (*2*–*4*). Apparently magnetized volcanic units suggest that a global field may instead have persisted as late at 3.8 to 3.6 Ga (*5*–*7*), although tying the ages of surfaces to that of possibly deep magnetization remains challenging.

The difference between these proposed timelines may have important implications for martian climate and habitability. Planetary dynamos have a complex relationship with atmospheric loss; although some escape pathways are independent of the global magnetic field, including neutral Jean’s escape and photochemical escape, which dominate modern atmospheric loss on Mars, the efficiency of ion escape varies with the intensity (*8*, *9*) and structure (*10*) of a planet’s magnetic field. While some field strengths and geometries may efficiently shield the atmosphere from solar wind and cosmic rays, others may instead accelerate atmospheric escape (*8*). The dynamo’s strength, structure, and longevity are therefore closely tied to the onset of large-scale climate change on Mars, making the planet’s magnetic history a key piece of the martian habitability puzzle.

The cessation age of the martian dynamo may also constrain Mars’ thermal history and interior properties. Recent work has shown that the lifetime of a thermally driven martian dynamo is sensitive to the initial core-mantle boundary temperature, core thermal conductivity, and mantle viscosity (*11*). If the dynamo was also partially driven by core crystallization, its history could additionally provide insight into the light element content of Mars’ core (*11*).

Another important question is whether the martian dynamo experienced polarity reversals. Although a number of authors have argued that reversals occurred on the basis of inversions of crustal magnetic fields (*5*, *12*, *13*), interpretation of crustal fields carries substantial uncertainties due to the inherent nonuniqueness of these inversions compounded by the unknown geometries and properties of the magnetized source regions. Because reversing dynamos are thought to characterize a restricted set of core Rayleigh numbers and to depend on the distribution of heating within the body and thermal and material properties of the core (*14*, *15*), determining whether the martian dynamo reversed could ultimately constrain Mars’ thermal history.

Because martian rock samples can be radiometrically dated and studied using laboratory paleointensity techniques, they may place precise constraints on the dynamo’s history that cannot be recovered from orbital data. Martian meteorites provide the best opportunity to study such samples in the absence of successful sample return missions. Unfortunately, strong, late shock events on Mars and magnetic overprinting on Earth disqualify most martian meteorites from retaining direct records of the martian dynamo (*16*–*20*). A notable exception is the orthopyroxenite meteorite Allan Hills 84001 (ALH 84001), which crystallized 4.091 ± 0.030 Ga ago on Mars (*21*). Excluding a thin fusion crust and a small (~1.5 mm) adjacent baked zone, the remanence hosted by ALH 84001 is of extraterrestrial origin [see below and (*22*, *23*)]. Because it escaped late severe shocks on Mars and remagnetization on Earth (*24*–*26*), it is the most likely known sample to still host ancient magnetic remanence. The paleomagnetic record of ALH 84001 may therefore be key to answering many of the remaining fundamental questions about the martian dynamo.

**Fig. 1.**
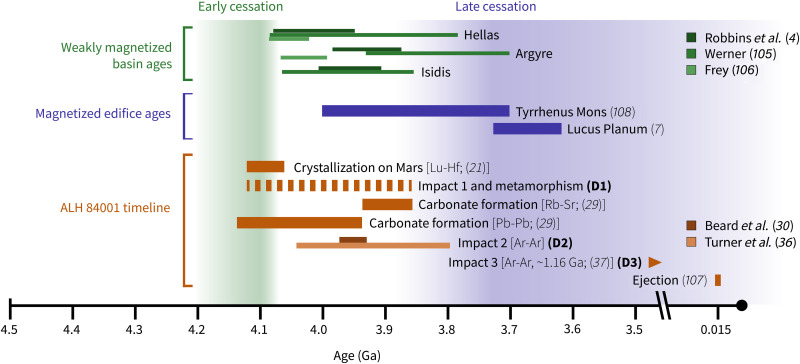
Timeline of constraints on the martian core dynamo and of events in the geologic history of ALH 84001. Young, magnetized edifices suggest the presence of a late strong dynamo (*7*, *108*), while weakly magnetized and demagnetized basins (*4*, *105*, *106*) are often interpreted as evidence of a weak surface field at 4.1 to 4.0 Ga. The labeling of impacts and metamorphisms as D1 to D3 reflects the convention established by Treiman (*24*), though the labels of individual events may differ from that work to reflect recent advances in the understanding of ALH 84001’s complex history (see the main text and section S6 for further discussion).

Two major ferromagnetic populations in ALH 84001 carry paleomagnetic records: magnetite- and pyrrhotite-bearing carbonates and pyrrhotite-bearing chromite-sulfide assemblages (*27*). The carbonates are by far the more studied of the two because they dominate the magnetization of bulk samples and are more easily resolved in magnetic field mapping. They likely precipitated from a low-temperature carbonate-rich fluid at 3.95 ± 0.02 Ga (*28*–*30*), although this origin is disputed (*31*, *32*). While it has been suggested that magnetite in the carbonate rims is biogenic (*33*), it is now most commonly attributed to siderite decomposition during an impact event (*34*, *35*). The ^40^Ar/^39^Ar thermochronology of ALH 84001 suggests that the last heating event that may have triggered this siderite decomposition occurred at 4.05 to 3.80 Ga, establishing an upper bound for the age of carbonate-hosted magnetization (*36*, *37*). Some samples also display an ^40^Ar/^39^Ar plateau at 1.16 ± 0.11 Ga (*37*), suggesting that another heating event may have occurred at that time. We term these two events D2 and D3, respectively, following the convention established in Treiman (*24*) and updated in Treiman (*38*) (Fig. 1; see section S6). Alternatively, this magnetite may have precipitated from low-temperature fluids during or after carbonate formation (*39*). Because of uncertainty in the peak temperatures of these events and in the relative timing of heating and magnetite crystallization, the carbonates may host a thermal or chemical remanent magnetization (TRM or CRM) of uncertain age.

Magnetic signals are also associated with some chromite-sulfide assemblages (*27*). While the chromite in ALH 84001 is expected to be paramagnetic at room temperature, pyrrhotite identified near and within chromite grains [see section S2; (*27*)] can carry the observed room-temperature remanent magnetization (*27*). Enrichments in some platinum-group elements support an igneous origin of the Fe-sulfides (see section S2.2), suggesting that they should have been initially magnetized upon igneous cooling and then fully remagnetized by high-grade thermal metamorphism during what we term the D1 event. This event is not independently dated but must fall between the rock’s crystallization at 4.091 ± 0.030 Ga and precipitation of the carbonates at 3.95 ± 0.02 Ga. Magnetization of the chromite-sulfide assemblages may have again been fully or partially reset during the D2 and D3 events, which attained peak shock pressures as high as 45 GPa (*40*, *41*). Some chromite-sulfide assemblages may have escaped remagnetization since D1 if they avoided recrystallization and remagnetization during the carbonate-forming event [see section S2 and (*41*)] and if the temperature and pressure fields of later impacts were sufficiently heterogeneous. This history suggests that chromite-sulfide assemblages may retain the oldest remanence in the meteorite—potentially as old as 4.1 Ga—but subpopulations may have been magnetized at least as recently as 3.9 to 3.8 Ga during the D2 event or ~1.16 Ga during the proposed D3 event.

Previous paleomagnetic studies of the carbonates and bulk samples have observed a pattern of locally unidirectional magnetization that becomes incoherent above millimeter scales (*22*, *23*, *27*, *42*, *43*). This pattern is not expected for igneous rocks cooled in a stable dynamo field, prompting speculation about other potential origins of the magnetization in ALH 84001. Several authors initially suggested that it could reflect impact-induced brecciation and clast rotation at centimeter scales such that all of the magnetization may have originally been acquired in a single heating event (*23*, *42*, *44*). However, nonuniform magnetizations were later observed at 100-μm scales in regions with no evidence for mutual rotation, suggesting that this pattern was produced in at least two unique magnetization events (*27*). One possibility that has been proposed in previous work is that nonuniform postimpact temperature and pressure conditions could have remagnetized subvolumes of the rock during times of distinct local field orientation, resulting in multiple magnetization directions (*22*, *23*, *37*). We refer to this as the heterogeneous impact remagnetization hypothesis.

The chromite-sulfide assemblages may provide a key test of this concept. As discussed above, their ancient age implies that their paleomagnetic record may be more complete compared to that of the younger carbonates, which also may not have initially acquired a TRM. Unfortunately, weak magnetizations and close proximities to other sources have made the magnetizations of most chromite-sulfide assemblages difficult to resolve with the bulk magnetometers and superconducting quantum interference device microscopes used in prior studies (*22*, *23*, *27*, *42*, *43*). The recently developed quantum diamond microscope (QDM) is a magnetic field imager with micrometer-scale resolution, which is sufficient to isolate the magnetic signals of individual chromite inclusions. The QDM can therefore be used to recover the direction and intensity of ancient martian magnetic fields recorded by individual ALH 84001 chromite-sulfide assemblages.

**Fig. 2.**
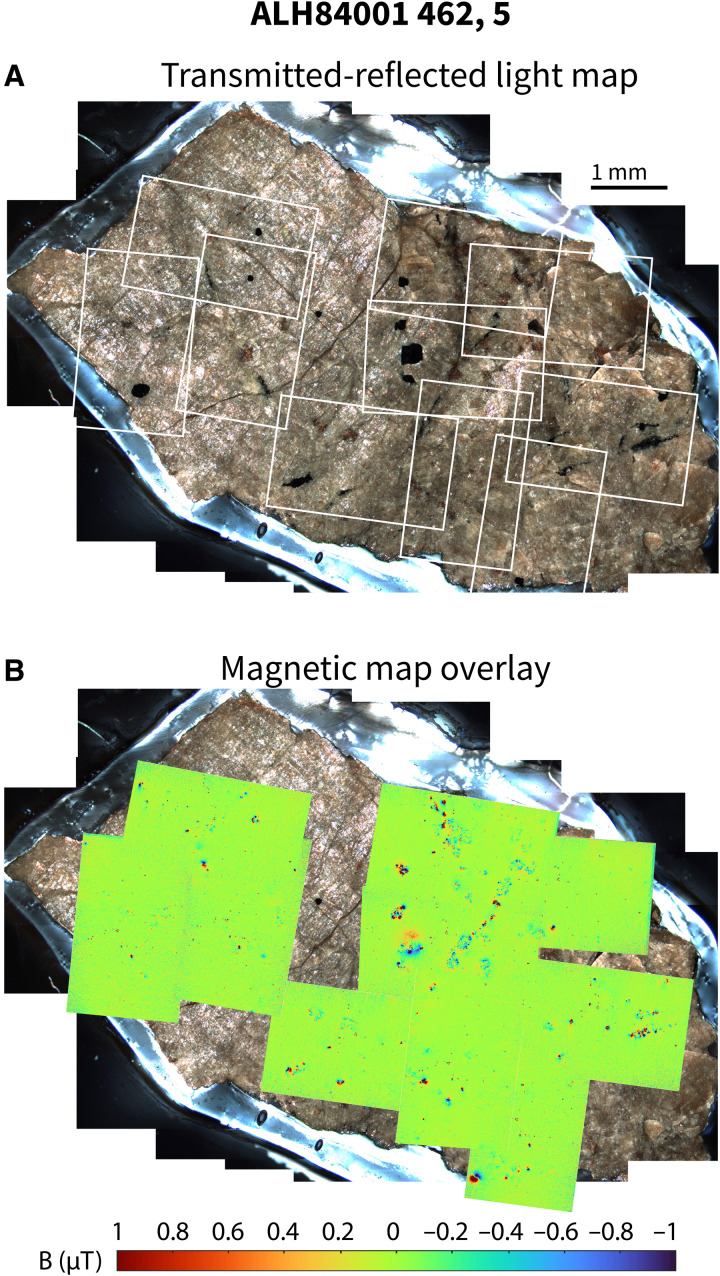
Summary of magnetic field imaging data. (**A**) Visible light map of the slice ALH 84001 462,5 studied in this work. The chromite-sulfide assemblages in this slice are visible as black euhedral grains and stringers. (**B**) Magnetic field map of ALH 84001 462,5. Green regions are areas of low magnetic field, while red and blue regions have strong negative (into the page) and positive (out of the page) magnetic fields, respectively. Many of the strong magnetic features visible in this map are associated with chromite-sulfide assemblages.

**Fig. 3.**
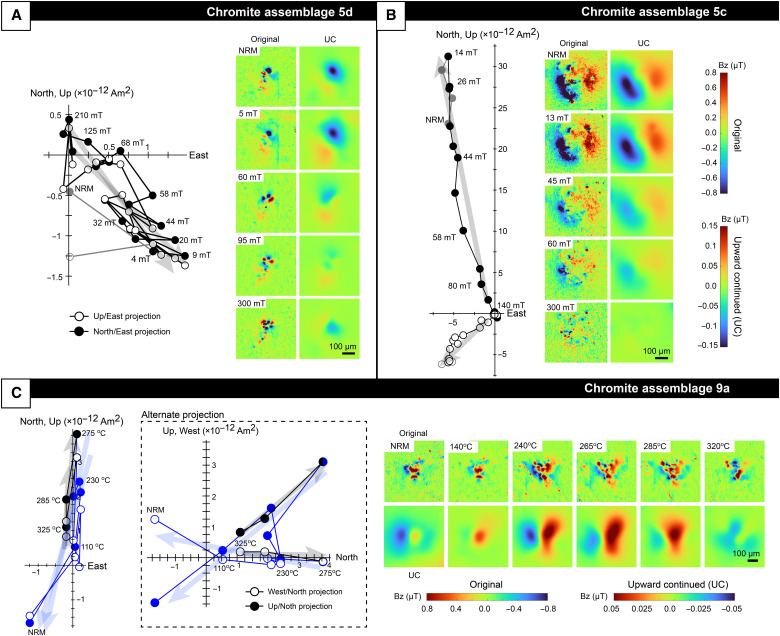
Orthogonal projection or Zijderveld diagrams and QDM magnetic field maps from demagnetization of NRM for three example sources. (**A** and **B**) AF demagnetized sources. Open and closed points in these panels represent projections onto the Up-East and North-East planes, respectively. The highest coercivity component is shown in black. Gray points are not included in any component. Maps of the raw magnetic field data are shown on the left and maps upward continued (UC) by 50 μm are shown on the right. (**C**) Thermally demagnetized source. An alternate projection is included in the dashed box for clarity, where open and closed points represent projections onto the Up-North and West-North planes, respectively. Black and blue points denote high- and low-temperature components, respectively. Original maps are shown at the top and maps upward continued by 100 μm are shown at the bottom. Higher demagnetization steps represent averages of multiple demagnetization steps to suppress noise. NRM measurements and 275° and 285°C steps in (C) (representing beginning of high temperature component) are not averaged. All sources were demagnetized to 300 mT/340°C, but steps are only shown until sources lose directional coherence and begin to produce unreliable and chaotic fit results. See table S1 and fig. S1 for fit details and Zijderveld diagrams of sources not shown here.

In this work, we take advantage of the QDM’s high spatial resolution to characterize the natural remanent magnetization (NRM) of chromite-sulfide assemblages in ALH 84001 (Figs. 2 and (3) under both alternating field (AF) and thermal demagnetization. We identified 10 chromite-sulfide assemblages exhibiting 12 total components of magnetization falling into two statistically significant, nearly antipodal directional clusters. This supports the heterogeneous impact remagnetization hypothesis, which predicts clustered NRM component directions corresponding to distinct heating events. We also identified a subpopulation of chromite-sulfide assemblages that appears to be very weakly magnetized, possibly due to heterogeneous impact remagnetization in weak crustal fields after the dynamo’s cessation. Overall, these results favor a martian dynamo that ceased later than 3.9 Ga and potentially experienced polarity reversals.

## RESULTS

### Paleomagnetism

**Fig. 4.**
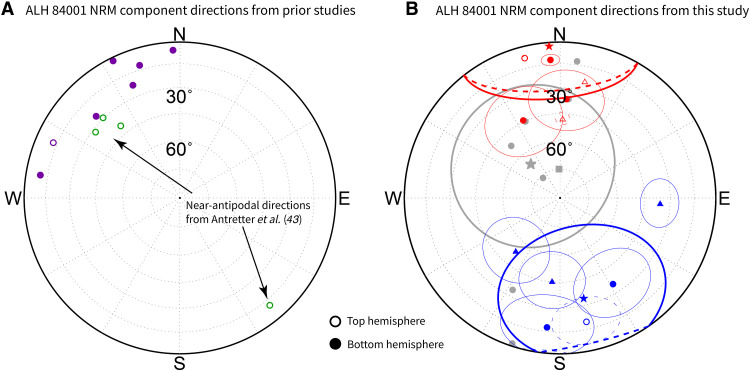
NRM component directions observed in ALH 84001 in this study and in previous studies. (**A**) NRM components calculated from demagnetization of ALH 84001 chips and bulk samples published in Kirschvink *et al.* (*42*) (purple) and Antretter *et al.* (*43*) (green). (**B**) NRM components identified in ALH 84001’s chromite population in this study; points falling in clusters A and B are shown in red and blue, respectively, with fusion crust directions shown in gray. Triangular points denote thermally demagnetized sources and circular points denote AF demagnetized sources. Stars represent best-fit mean directions for each cluster. The gray square shows the high-temperature component direction recovered from thermal demagnetization of a fusion crust sample. Fit MAD values are plotted as thin error circles around each individual data point and ɑ_95_ regions of each cluster are plotted as thick solid lines. Data from different studies are not mutually oriented with respect to each other. Note the clustering observed in all studies and the possible antipodal components recovered in Antretter *et al.* (*43*).

To isolate stable magnetization components, we demagnetized three mutually oriented, 100- to 300-μm-thick polished slices of the sample ALH 84001,462. Each slice measured approximately 1 cm by 0.5 cm. We subjected the two slices 462,5 and 462,10 to stepwise three-axis AF demagnetization up to 300 mT and the slice 462,9 to thermal demagnetization up to 340°C, upon which all magnetization components in chromite-sulfide assemblages were removed (Fig. 3). Analyzing the resulting demagnetization sequences for each source using principal components analysis (*45*) yielded 10 total chromite-sulfide assemblages that hosted robust components. Seven of these were found in the AF demagnetized slices 462,5 and 462,10 and carried high-coercivity components blocked up to 52 to 240 mT [Figs. 3 (A and B) and 4B, round red and blue points]. The thermally demagnetized slice 462,9 hosted the remaining three chromite-sulfide assemblages, which contained five total high-temperature components blocked up to 275° to 340°C (Figs. 3C and 4B, triangular red and blue points). All components contained at least three consecutive demagnetization steps with some AF components containing up to 34 steps, some of which were repeated applications of the same AF level to reduce noise. Included components had a maximum angular deviation (MAD), which quantifies the confidence of the fitted component direction, of ≤20°.

To reduce the risk of characterizing a late overprint, including one acquired viscously on Earth, we considered only components that persisted at least partially above 50 mT during AF demagnetization. For thermally demagnetized sources, we considered only components persisting above the 110°C step because material unblocked above that temperature during laboratory demagnetization would require ~50°C temperatures during the entire 15,000-year residence in Antarctica to represent a viscous remanent magnetization (VRM) acquired on Earth (*23*). Similarly, ~40°C temperatures during 4 Ga on Mars would cause viscous decay of magnetization that would unblock at 110°C in laboratory demagnetization (*23*). As a result, low-temperature components identified in two thermally demagnetized sources cannot be VRM and are therefore included in our analysis. The NRM magnetic moments of these chromite-sulfide assemblages were on the order of 10^−13^ to 10^−12^ Am^2^, corresponding to a ~5° directional error for a 50-μT magnetizing field and ~10° for a 5-μT magnetizing field resulting from the statistical limit of paleomagnetic recording (*46*).

Last, we observed a set of four chromite-sulfide assemblages that did not display a quantifiable NRM direction but were capable of acquiring substantial anhysteretic remanent magnetization (ARM), which is a room temperature laboratory analog of a TRM (*47*). These sources are therefore viable paleomagnetic recorders that carried no NRM component due to remagnetization in a weak magnetic field (see section S5.2 and fig. S13). Some carbonates also display this behavior; because thermal demagnetization in previous studies suggest that carbonate-hosted magnetite can be blocked up to 580°C and does not acquire substantial VRM (*22*), the weak magnetization of these sources may have been acquired during the same heating event that produced the weak NRM in some chromite-sulfide assemblages. Magnetic sources with this property have not been previously reported in ALH 84001.

Analysis of AF and thermal demagnetization of NRM for the 10 chromite-sulfide assemblages with coherent magnetizations revealed that the magnetization components fall into two directional clusters (Fig. 4B). Anisotropy correction (see Materials and Methods) shifted the mean direction of each cluster by 4° and 6° to corrected values of *D* = 356° and *I* = 2° for cluster A and *D* = 167° and *I* = 34° for cluster B, where *D* and *I* denote declination and inclination. These corrected clusters had ɑ_95_ values of 34° and 40° and precision parameters *k* of 4.8 and 3.8. Neither of these cluster directions is similar to the steep mean direction of *D* = 319° and *I* = 66° extracted from fusion crust in the sample (Fig. 4B, gray points), suggesting that these magnetization components were not acquired during or after Earth atmospheric entry (see section S1).

The clusters in the original and anisotropy-corrected datasets are separated by 150° and 142°, respectively. An analytical reversal test (*48*) on these clusters yielded an indeterminate result, indicating that the clusters are consistent with an ideal dipole reversal but cannot be formally classified as one due to the large uncertainties in cluster mean directions. We confirmed this result using a parametric bootstrap reversal test assuming each cluster was Fisher distributed (*49*), which showed that the mean of one cluster cannot be statistically distinguished from the antipode of the mean of the other. Notably, Antretter *et al.* (*43*) recovered two similarly antipodal directions separated by ~130° from the low- and medium-coercivity NRM components of a bulk sample (Fig. 4A, green points).

### Cluster significance testing

Because the directional clustering we observe has important implications for the origin of magnetization in ALH 84001 chromite-sulfide assemblages, we carefully consider whether the observed clusters A and B may have arisen through random chance. Each cluster is individually statistically inconsistent with randomness according to a classic paleomagnetic conglomerate test (*50*). However, the relatively small total number of data points and the manual assignment of cluster membership led us to also consider whether the full dataset is consistent with a random distribution. To assess this, we tested whether random sampled directions could produce clusters statistically similar to those in our dataset. We first randomly generated 20,000 sets of 11 directions and used *k*-means clustering to separate each dataset into the two best-defined clusters. This procedure, which reproduced our manually assigned clusters in the experimental dataset, simulates the division of a random set of directions into two clusters by visual inspection. We then calculated the mean and precision parameter *k* of each assigned cluster.

We identified three ways in which our experimental dataset should differ from these randomly generated directional clusters to be considered statistically inconsistent with randomness:

1) The *k* value for each individual cluster should be large, reflecting tight grouping of source directions.

2) The minimum *k* between the two clusters should be large compared to those in the random datasets, reflecting the existence of two true directional clusters instead of a single tight cluster plus a collection of scattered residual directions.

3) The *k* of the two clusters should be similar, reflecting formation and dispersion of both clusters by the same physical processes.

The *k* values of the tighter and looser clusters were larger than those of 93 and 83% of all clusters in the random dataset, respectively, and the minimum *k* value between the two clusters exceeded the minimum *k* value in 98% of random samples. The two clusters had *k* values more similar to one another than 63% of random samples. Because these three properties are not necessarily independent, we cannot explicitly calculate a single probability that our data are nonrandomly dispersed. However, our dataset achieved 98% significance on one of the three measures and is positioned on the tails of all three distributions, affirming that it is highly improbable to have arisen from random magnetizations. This supports our identification of two directional clusters.

### Magnetization mechanism

We consider how chromite-sulfide assemblages, sometimes separated at <1-mm scales, acquired magnetizations in distinct grouped directions. Nearly all the sources for which we could compute ARM anisotropy tensors were slightly or moderately anisotropic (1.4 < *P′* < 2.9) (*51*). The corresponding corrections to the fitted NRM directions were all ≤10° with the exception of one source corrected by 20°. There was no clear global easy axis among the anisotropy tensors of measured sources; therefore, sample anisotropy cannot account for either of the clusters in stable component directions.

Another possibility is that the observed pattern of magnetization, which forms two nearly antipodal clusters, was produced by magnetic self-reversal or rotation within the sample. The former is highly unlikely because self-reversal in pyrrhotite has been rarely reported and requires magnetostatic interaction with a higher Curie temperature phase (*52*). This condition is not met in ALH 84001 chromite-sulfide assemblages (see section S2). Postmagnetization rotation at the subsample scale is also unlikely to have produced the observed pattern because there is no evidence for large mutual rotations within ALH 84001,462 after the D1 event that produced the chromite stringers. Larger-scale rotation of the entire rock or block remains a possibility and will be further addressed in Discussion.

As discussed above, heterogeneous impact remagnetization represents a proposed mechanism by which ALH 84001 chromite-sulfide assemblages may retain a record of multiple magnetizing events, potentially resulting in two observed directional clusters. To test whether this process can quantitatively reproduce the observations, North *et al.* (*41*) performed mesoscale shock simulations on several regions of ALH 84001,462 and modeled fine-scale variations in pressure and temperature.

Most of the postshock thermal heterogeneity in these simulations was driven by mineralogical differences and the development of shear zones. Chromite experienced <100°C direct postshock heating for shocks up to 50 GPa while carbonate under the same conditions reached temperatures well over 1000°C. Therefore, chromite-sulfide assemblages located near carbonate or regions of high shear could have been preferentially thermally remagnetized. Because equilibrium temperatures remained below 320°C for shocks with average bulk pressures below ~45 GPa—the highest pressure estimated for the D2 or D3 shock events—postimpact thermal equilibration would not have fully demagnetized the chromite-sulfide assemblages located away from strong heat sources. These mesoscale impact simulation results are therefore consistent with the heterogeneous magnetizations we observe. Furthermore, some chromite-sulfide assemblages in this scenario could have remained below the Curie temperature of pyrrhotite while high temperatures were simultaneously reached elsewhere to initiate siderite decomposition in the carbonates (*34*).

Our paleomagnetic results also require temperatures during the D3 event at 1.16 ± 0.11 Ga to have been much lower than some previous estimates. Peak temperatures of ~1400°C were proposed for this event to account for preferential degassing of orthopyroxene over maskelynite, assuming that the two phases were heated to the same temperature (*37*). However, systematic differences in the heating of orthopyroxene and maskelynite may allow the same preferential degassing to occur with less intense heating. North *et al.* (*41*) demonstrated that the peak temperatures of 1050°C in orthopyroxene and 900°C in maskelynite produced during a 45-GPa impact event could yield the observed local preferential degassing of orthopyroxene with an equilibrium temperature of ~300°C. Our results also require that equilibrium temperatures during the ejection event (D4) and likely also the D2 event did not closely approach the pyrrhotite Neél temperature of ~320°C, consistent with existing pressure and temperature constraints for these events (see section S6).

The effect of shock pressure during these impacts on remanence remains uncertain. Their estimated peak pressures are higher than values associated with phase transitions in pyrrhotite at 2 to 5 GPa, which have been shown to result in remagnetization during small shocks (*53*, *54*). However, because we observe two nonrandom directional clusters, we can infer that pressure during a single impact event could not have fully remagnetized all chromite-sulfide assemblages. Because even the ejection event exposed ALH 84001 to pressures uniformly above 5 GPa, some chromite-sulfide assemblages must have avoided remagnetization by a pyrrhotite phase transition. This is consistent with laboratory results indicating that pyrrhotite may retain a large fraction of its preshock remanence through shocks as high as 4 to 12 GPa, potentially due to heterogeneity in the pressure field or distinct polymorphs of pyrrhotite (*53*). In summary, although some previous experiments suggest that there is a phase transition in pyrrhotite at low pressures, our observations imply that remanence acquired during the D1 and D2 events survived the high pressures experienced during subsequent impacts.

A final uncertainty is whether chromite-hosted sulfides may have been chemically altered during the carbonate-forming event. Because the sulfides are enriched in nonmobile Pt-group elements and many are euhedral, the sulfides must be igneous with no alteration more extensive than partial or full in situ recrystallization (see section S2). However, alteration-prone sulfides located on chromite exteriors or in cracks are chemically identical to those in the unbroken interiors of some chromites, making even partial recrystallization improbable.

In the unlikely case that sulfides were substantially recrystallized during the carbonate-forming event, which is the only known aqueous alteration event in the rock’s history (*38*), our interpretation of the two observed magnetized populations would be essentially unchanged. If one population definitively hosted a CRM, we could precisely date the earlier magnetization to 3.95 ± 0.02 Ga. However, a 3.95 ± 0.02 Ga recrystallization event could instead have modified the carriers of preexisting TRM while preserving the original direction (*55*); in this case, the age of the surviving remanence would still correspond to the original TRM event, likely D1. The best estimate of the age of the older magnetization therefore remains unchanged at 4.1 to 3.95 Ga. Although the paleointensity calibration for a CRM would likely differ from typical TRM paleointensity calibration, the true value could be either higher or lower and would likely be within tens of percent of the inferred value (*55*). Because the available evidence suggests that the sulfides were not substantially recrystallized after the D1 event and because assuming they carry TRM would still give a reliable paleointensity estimate, we assume that the sulfide remanences we observe are thermal.

### Paleointensities

On the basis of the above discussion, we estimate TRM paleointensities for each component. We AF demagnetized a 300-mT AC field, 200-μT DC field ARM to estimate single-source paleointensities for sources in slices 462,5 and 462,10 and performed stepwise partial thermoremanent magnetization (pTRM) acquisition up to 340°C in a 200-μT northward field to estimate paleointensities for sources in slice 462,9. To limit the effects of potential alteration, we did not perform a traditional Thellier-Thellier dual heating experiment for thermally demagnetized sources: Instead, pTRM acquisition experiments were performed after thermal NRM demagnetization analysis was fully completed. Because the samples were exposed only to weak (5 to 20 nT) residual oven fields during NRM demagnetization, the recovered components could not have been acquired via alteration and magnetization during heating.

Clusters A and B sources had respective average estimated paleointensities of 42 ± 20 μT and 15 ± 11 μT after correction for difference in ARM and TRM efficiency (see Materials and Methods). The overall average paleointensity was 28 ± 13 μT. Much of the uncertainty in our single-source paleointensity estimates can be attributed to uncertainty in the ARM to TRM correction factor *f′*. These values are similar to previous paleointensity estimates of ALH 84001 chromite-sulfide assemblages calculated using the saturation isothermal remanent magnetization (sIRM) paleointensity method (*27*) and support prior findings that fields near Mars’ surface were of similar strength to surface fields on Earth.

In addition to the clustered directions discussed above, we identified four chromite-sulfide assemblages that exhibited weak NRM magnetic field signals with much lower net magnetization than the same sources after imparting 10- to 20-μT DC field ARMs (see section S5.2). After accounting for the different efficiencies of ARM and TRM, this implies that these sources were magnetized in a <6-μT field, likely after the dynamo’s cessation.

## DISCUSSION

### Magnetization origin

To summarize, we have identified three populations of chromite-sulfide assemblages in ALH 84001: two strongly magnetized groups that record distinct and nonrandom magnetization directions separated by 142° and one weakly magnetic group that was most recently demagnetized in a <6-μT magnetic field. The two strongly magnetized populations were magnetized in the presence of 42 ± 20 μT and 15 ± 11 μT fields, respectively. After considering several mechanisms for generating locally heterogeneous magnetization, we argued that heating in at least three distinct impact events, two of which occurred in substantial magnetic fields, is the most probable explanation for the observed magnetization groupings. We now consider when and in what ambient magnetic fields these remanences may have been acquired.

We first consider whether ALH 84001 chromite-sulfide assemblages record transient impact-generated magnetic fields produced in ionized vapor above the planetary surface (*56*). It is unlikely that either individual directional cluster represents a record of an impact-generated field because such fields are predicted to be >100 μT—exceeding observed paleointensities—in the regions of impact structures where heating exceeds >320°C (*56*). Impact-generated plasma fields are therefore unlikely to have produced the observed pattern of magnetization. The apparent lack of such impact-generated fields is potentially due to effective quenching of ionization in a thick martian atmosphere, believed to have had a surface pressure of at least 0.5 bar during the Noachian (*57*). Such high pressures would imply that multiple paleomagnetic studies showing the lack of impact-generated magnetic fields around terrestrial craters are relevant to early martian conditions (*58*, *59*).

Ruling out such ephemeral magnetic fields implies that ALH 84001 chromite-sulfide assemblages recorded long-lived ambient magnetic fields at two distinct times. In this case, the ambient magnetic field must have rotated ~140° relative to the ALH 84001 meteorite precursor material between magnetization events. We consider two possibilities for the origin of this rotation.

**Fig. 5.**
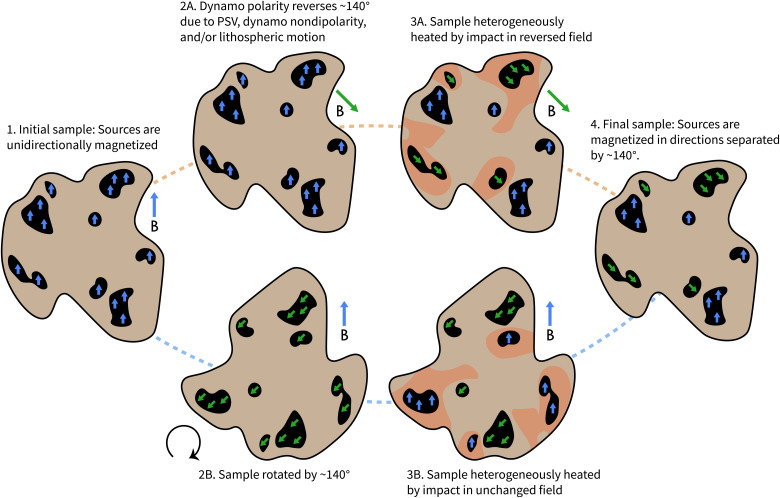
The two pathways by which ALH 84001 chromite-sulfide assemblages (dark regions) may have acquired two nearly antipodal groups of magnetization. Both processes rely on the heterogeneity of impact heating to remagnetize only a subset of the sources. Because the probability of impact-related brecciation rotating the sample by such a large angle is small, we favor the top process, which implies that the martian dynamo experienced at least one polarity reversal.

First, the observed nearly antipodal directional clusters may have been produced by heterogeneous remagnetization following ~140° rotation of the entire centimeter-scale clast, possibly as part of a much larger crustal block (Fig. 5, bottom). The endmember assumption that all clast rotation angles are equally likely yields a maximum probability of 0.22 that the directions before and after rotation would be within 38° of antipodal as observed (see section S4). Because smaller angle rotations of a clast are more probable, and large structural rotations are restricted to small regions of an impact structure (*60*), the true probability of an impact producing such a large rotation is likely substantially lower.

A second, more likely possibility is that ALH 84001 was heterogeneously remagnetized following a martian dynamo polarity reversal (Fig. 5, top). This scenario, which is consistent with evidence for reversals from paleopole inversion studies (*5*, *12*, *13*), would naturally reproduce the large angular separation between the two directional clusters as well as their Earth-like paleointensities. However, a key challenge for conclusively preferring this model is that the expected signature of a martian reversal recorded in ALH 84001 is not well understood. We identify and examine three sources of uncertainty.

First, the nearly instantaneous magnetization acquisition during postimpact heating implies that ALH 84001 chromite-sulfide assemblages do not average any paleosecular variation (PSV). Because the nature of martian PSV is unconstrained, this introduces a random perturbation of unknown amplitude to the angular separation between two reversed directions. Assuming Earth-like PSV characterized by a Fisherian distribution with a precision parameter of κ = 25 (*61*), two reversed directions drawn at random have just a ~3% chance of being ≥38° from antipodal. However, accounting for uncertainty in the cluster center locations and the corresponding uncertainty in reversal asymmetry yields a much larger probability of ~10% that Earth-like PSV alone could account for the observed deviation of the cluster separation from antipodal. This suggests that a polarity reversal of a martian dynamo with Earth-like PSV could produce the observed dataset.

Second, the degree of reversal asymmetry for a martian dynamo, which is related to the balance between dipole and higher order terms, is unknown. Because higher relative quadrupole and octupole components can yield greater reversal asymmetry, a martian dynamo with a smaller relative dipole component than Earth could produce less locally antipodal reversals. We find that our results are consistent with a broad range of field geometries given the uncertainty in cluster means (see section S7).

Geophysical or tectonic factors could also have caused reversed magnetizations to deviate from antipodal directions. The formation of Tharsis, for example, may have produced true polar wander (TPW) of up to 60° (*62*, *63*) potentially between the D1 and D2 events (*64*). Subantipodal paleopole clusters reported in studies of martian crustal fields also suggest that TPW and reversal events may have occurred contemporaneously (*12*, *13*, *65*). Although this TPW could not alone produce the ~140° angular separation between the clusters, it could produce an asymmetric reversal record if it occurred between impact heating events. Minor brecciation and rotation between magnetization events could also cause a reversal pattern to deviate from antipodality or enhance existing deviation.

A third and final possibility is that the older and younger of the two directional clusters record dynamo and crustal magnetic fields, respectively. However, as discussed in greater detail below, this scenario would require anomalously strong local crustal magnetic fields, and a substantial change in the crustal field between D2 and D3 to explain the presence of the unmagnetized source population.

Combined, these effects prevent us from precisely predicting the angular separation between recorded reversed directions for an early martian dynamo. Therefore, we cannot quantitatively assess the likelihood of the dynamo reversal model and compare it to that of a large clast rotation. However, given the large, 142° angular separation between the two directional clusters, the broad set of plausible mechanisms that would cause a recorded reversal to deviate from antipodality, and the low probability of such a large rotation resulting from an impact event, we suggest that heterogeneous impact remagnetization following a martian reversal is the most likely origin of the magnetization directions in ALH 84001 chromite-sulfide assemblages.

Even if our experimental methods can be extended to a broader population of chromite-sulfide assemblages in ALH 84001, the inherently limited sampling of the martian dynamo within this single meteorite implies that the above uncertainties would remain. Furthermore, documented shock brecciation and rotation at centimeter scales within the sample (*42*) may limit the number of chromite-sulfide assemblages for which component directions can be meaningfully statistically grouped, limiting the volume even within ALH 84001 that can be used for paleomagnetic direction analysis. Additional samples, potentially from martian sample return missions, will likely be required to conclusively resolve the martian reversal question.

### Magnetization timing

Regardless of whether the magnetizations record a reversal or rotation event, the existence of two directional clusters imply that ALH 84001 chromite-sulfide assemblages were magnetized in at least two distinct episodes. We consider the possible timings of these magnetization events given the known history of ALH 84001 (Fig. 1).

In the first scenario, one of the two directions would have been acquired during the D1 event and could be as old as 4.1 Ga. The other would then date to the D2 event, postdating carbonate formation at 3.95 ± 0.02 Ga (*29*). This interpretation is supported by Ar-Ar thermochronology and textural observations indicating that D1 and D2 represent the two strongest impact events in the history of ALH 84001 (see section S6). Furthermore, the mineralogically estimated peak pressure of D2 is consistent with heterogeneous thermal remagnetization based on mesoscale impact simulations (*41*). After the dynamo’s cessation, the D3 event at 1.16 ± 0.11 Ga would have again heterogeneously remagnetized a separate group of chromite-sulfide assemblages in crustal fields no stronger than 6 μT to produce the weakly magnetized population. This interpretation is consistent with the ~5-μT paleointensity recovered from the Nakhlite MIL 03346, which is thought to record martian crustal fields (*20*) as well as with the low crustal field strengths inferred from other martian meteorites and directly measured by the InSight lander (*19*, *66*).

The implications of this scenario for Mars’ magnetic history depend on whether the strongly magnetized populations record dynamo or crustal fields. Although a crustal origin for both magnetizations is unlikely due to the low probability of a near-antipodal rotation, as discussed above, the younger directional cluster could, in principle, record a crustal magnetic field at ~3.9 Ga. This would still imply that the dynamo was active until at least 4.1 to 3.95 Ga but not necessarily reversing. To assess this possibility, we first consider whether the clusters’ paleointensities are consistent with the strengths of crustal fields on Mars. The lower of the two paleointensities is at least 2 to 10 times stronger than the paleointensities recovered from younger martian meteorites, which must have been magnetized after the dynamo’s cessation (*19*). However, crustal fields vary considerably across the martian surface (*67*). Assuming that field strengths at the surface are 100 times stronger than those measured at 200-km altitude, consistent with measurements from InSight (*66*), we can estimate surface field strengths from models of martian crustal fields at 200 km altitude. If the distribution of crustal fields today is an appropriate analog for those at ~3.9 Ga, we would expect 4% of surface fields to exceed the lower paleointensity estimate of 15 μT. Including the uncertainty in our paleointensity estimate yields a 9% probability that ALH 84001 crustal fields could produce the magnetization intensity observed in the southerly cluster (fig. S10). Paleointensity alone therefore implies that a crustal field record at D2 time is unlikely but cannot be conclusively rejected.

More informatively, the presence of an even more weakly magnetized population that was demagnetized in a ≤6-μT ambient magnetic field during the D3 event implies that ALH 84001 was situated in a region with relatively weak crustal fields by 1.16 Ga. Therefore, for one of the strongly magnetized populations to also record crustal fields, the local field strength must have decreased substantially between D2 and D3. We regard the highly specific sequence of events necessary to magnetize two of the observed populations in crustal fields as ultimately improbable.

In contrast, if both clusters record dynamo fields, there are a number of other mechanisms that could account for the difference in their paleointensities. Most straightforwardly, the difference could be caused by variation in the global field’s local moment due to global nondipolarity or interaction between a varying dynamo field and a constant crustal one. The dynamo could also have experienced a change in overall intensity between the two magnetization events. In any case, because both paleointensities reported here are within the range typically reported from Earth samples (*68*) and we might expect greater variation from a less-dipolar martian dynamo (*69*), the difference between them is consistent with a dynamo origin.

As a second scenario, the two directions may have been magnetized during the D2 and D3 impact events and record dynamo fields at 4.0 to 3.9 Ga and crustal fields at 1.16 ± 0.11 Ga. In this scenario, the weakly magnetized chromite-sulfide population would be difficult to explain. The only additional known impact is the event that ejected ALH 84001 from the martian surface, which may not have produced sufficient heating to remagnetize chromite-sulfide assemblages [see section S6 and (*41*)]. If the weakly magnetized population was magnetized during the ejection event, then the crustal magnetic field in the vicinity of ALH 84001 must have decayed substantially between ~1.16 Ga and 15 Ma.

Because we consider it unlikely that either directional cluster records martian crustal fields for the reasons discussed previously, we consider the first timeline, which involves two strong, reversed fields recorded at 4.1 to 3.95 Ga and 3.9 to 3.8 Ga and partial demagnetization in a weak field at 1.16 Ga, more likely. This interpretation supports tentative evidence for martian reversals from analysis of martian crustal fields (*5*, *12*, *13*) and nearly antipodal magnetizations observed in one previous paleomagnetic study (*43*) but represents the first concrete evidence of martian reversals from a rock sample. Critically, both possible histories outlined above imply that one of the directional clusters records a dynamo field at ~3.9 Ga. This supports previous arguments for a long-lived dynamo but does not distinguish between different proposed late cessation ages (*5*–*7*). A dynamo persisting until 3.9 Ga or later may have far-reaching implications for the planet’s climatic and thermal evolution as well as for the interpretation of modern-day crustal fields on Mars.

### Implications of a long-lived, potentially reversing martian dynamo

Any dynamo persisting later than ~4.1 Ga challenges the interpretation that weakly magnetic large impact basins record a permanent dynamo cessation. If the dynamo was indeed long-lived, these weak fields could reflect variation in the depth or composition of the magnetized layer or excavation of this layer during impact (*7*). Alternatively, if the martian dynamo was reversing, basin cooling through multiple reversals may naturally produce weak magnetic fields above some large basins without requiring any field weakening or cessation (*70*, *71*).

A long-lived martian dynamo also has important implications for Mars’ thermal history. It has been argued from theoretical considerations that a thermal dynamo on Mars would have been extremely short-lived, with persistence of such a dynamo until even 4.1 Ga requiring an initial core temperature substantially hotter than the mantle (*72*) or a late jump in core-mantle boundary heat flux driven by changing mantle mineralogy (*73*). Hemingway and Driscoll (*11*) identified several further possible thermal histories that could permit a dynamo to persist until at least 4.1 Ga including higher initial core-mantle boundary temperatures and low mantle viscosities. However, that work and others assumed core conductivities much higher than the 5 to 30 W m^−1^ K^−1^ suggested by recent experiments (*74*). These revised conductivity estimates may permit a thermal dynamo to persist until 3.9 Ga or later, with the specific cessation age dependent on the particular values of the core conductivity and reference mantle viscosity (*75*).

Alternatively, core crystallization may have prolonged the lifetime of the martian dynamo. Recent results from the InSight mission favor a liquid martian core but cannot exclude a small solid inner core (*76*). A partially solid martian core would be consistent with a dynamo partially driven by core crystallization. Although the lack of present-day dynamo activity on Mars has been interpreted as evidence that the martian core has not begun to solidify, recent work suggests that a range of mechanisms could produce a partially solidified core without generating substantial core convection today (*11*). If a long-lived martian dynamo ultimately requires temporary compositional dynamo action, this would impose limits on the type and concentration of light elements in the martian core (*11*).

The long-lived dynamo implied by our results may also have important implications for the habitability of early Mars. Much of the change in Mars’ climate since early in the planet's history, when liquid water was prevalent on its surface, can be attributed to the loss of a large fraction of its atmosphere (*77*). The stability of liquid water at Mars’ surface during valley network formation suggests that it hosted an early CO_2_ atmosphere much denser than its modern atmosphere, possibly up to 1 to 2 bar (*78*). Gas trapped in ALH 84001—including that in the carbonate population—is largely unfractionated, implying that a major atmospheric escape did not begin before carbonate formation at 3.95 ± 0.02 Ga (*79*).

If Mars’ dynamo effectively shielded the atmosphere from erosion, its waning and cessation after 3.9 Ga may have triggered the planet’s change in climate. However, the overall impact of planetary magnetic fields on atmospheric escape is heavily debated, with some authors reporting that a global field on Mars could have minimal effect on (*80*) or increase (*8*, *9*) atmospheric escape rates. Further work to understand how Mars’ dynamo shaped its atmosphere will be necessary to determine the implications of a long-lived martian dynamo for surface conditions and habitability on ancient Mars.

## MATERIALS AND METHODS

### Sample and source selection

The 0.64-g ALH 84001,462 sample used in this study was obtained from the Johnson Space Center. It was selected in part due to the presence of fusion crust on one face, enabling a fusion crust baked contact test. From this sample, we produced 13 mutually oriented, 100- to 300-μm-thick polished slices. Three slices (462,5; 462,9; and 462,10; see fig. S3) were chosen for paleomagnetic study. From these, we selected 32 magnetically isolated chromite-sulfide assemblages between 50 and 500 μm in diameter for QDM mapping. We included both euhedral chromites and isolated sections of chromite “stringers” modified by shear during the D1 event as both populations are ancient and span the same compositional range (*24*). The distances between measured chromite-sulfide assemblages in each slice and the >500-μm separation between the imaged surfaces of adjacent slices (corresponding to the thickness of each slice plus the material removed by the 270-μm-thick nonmagnetic diamond-impregnated wire saw used to slice the sample) ensures that each analyzed source represents a unique chromite-sulfide assemblage. In one case, a longer chromite was cut into several smaller segments using a nonmagnetic dental tool to produce isolated magnetic sources that could be used for net moment analysis (*81*).

To identify and avoid contamination from magnetization acquired on Earth atmospheric entry, we performed a baked contact test using seven small segments of fusion crust in samples 462,6, 462,7, 462,8, and one large segment from 462,11. We found that the seven small fusion crust samples displayed moderate scatter, typical of fusion crust in ALH 84001 (*27*) and other meteorites (*82*), centered around a steep average NRM direction of *D* = 315° and *I* = 64° (Fig. 4B, gray points). Thermal demagnetization of the additional larger sample of fusion crust, measured with a 2G Enterprises Model 755 Superconducting Rock Magnetometer, yielded a high-temperature component ending at 550°C with a similar direction of *D* = 358° and *I* = 75° (see fig. S2). In total, these eight directions were clustered around an average direction of *D* = 319° and *I* = 66° with an ⍺_95_ of 43° [nonrandom with *P* < 0.01 in Watson test (*50*)]. Sources 1.5 mm away from the fusion crust did not exhibit this average direction, consistent with the ~1-mm baked zone previously documented in ALH 84001 (*22*, *23*). We accordingly restricted our study to chromites located at least 1.5 mm away from the fusion crust.

### Paleomagnetism

Stepwise three-axis AF demagnetization of the two slices 462,5 and 462,10 up to 300 mT occurred in 5- to 50-mT steps. We produced QDM magnetic maps of the NRM (e.g., Fig. 2) and of the remaining magnetization at each demagnetization step, mutually orienting all maps to within 1°. Applying a 0.9-mT QDM bias field that reversed twice during each imaging step yielded a residual effective bias field of ±400 nT during measurements. Because internal reflection within the sample produces global fluorescence that contributes to the measured optically detected magnetic resonance spectrum at each pixel (*83*), we corrected for this following the process detailed in Fu *et al.* (*81*), adopting a global fluorescence factor of 0.4. These corrections and much of the later analysis were performed using the QDMlab MATLAB toolbox (*84*).

After identifying the regions of each magnetic map that corresponded to chromite-sulfide assemblages visible on the surface of the slice, we cropped out each of these regions to exclude signal from nearby magnetic sources. Because the QDM sensor-to-sample distance is typically smaller than an individual chromite, their signals in the raw magnetic maps typically deviate from a dipole pattern (e.g., Figs. 2 and 3). We therefore upward continued maps by up to 75 μm until the fraction of signal that could be accounted for by a single dipole, also known as the dipolarity parameter, was at least 60% and more typically 60 to 65%. Previous analysis has shown that these dipolarity parameters correspond to a 10° to 15° angular uncertainty in the fitted direction (*81*). Upward continuation magnitude was permitted to vary between sources but was held constant for each source across all demagnetization steps.

We repeated this analysis for the slice 462,9, which was thermally demagnetized up to 340°C in steps of 10° to 40°C. Heating was performed in a 99.999% pure CO_2_ atmosphere, which has an oxygen fugacity similar to that of ALH 84001 (FMQ-2.7 log *f*O_2_) at low temperatures (*85*, *86*), to limit alteration during heating. We flushed the controlled atmosphere chamber with CO_2_ before beginning each heating and maintained a steady flow of 2 liters/min throughout. Selected thermal components met the same requirements on dipolarity parameter and MAD value imposed on AF components.

Although we initially selected 32 chromite-sulfide assemblages for study, only 10 of these were found to carry robust magnetization components. Four of the remaining 22 were classified as weakly magnetic as discussed above. Most of the remaining sources could not be reliably fitted due to geometrical limitations of our fitting methods (e.g., too large or too close to other sources) or insufficient magnetic content. Several could be fitted and demagnetized but produced components that did not pass the selection criteria; these sources may carry no coherent magnetization due to the lack of sufficient independent magnetic particles (*46*). Because the size, location, and concentration of sulfides varies substantially between chromites, and given the expected multiple heterogeneous heatings this meteorite was subjected to, it is unsurprising that some sources do not carry easily recoverable components.

### Anisotropy correction

To correct for source anisotropy, we computed the best-fit anisotropy of magnetic remanence (AMR) tensors ***K*** for each source from measurements of ARM along six axes or TRM along three axes. We determined the best-fit anisotropy tensor ***K*** for each individual source as the least-squares solution of

$M_{i}=K_{ij}H_{j}$ where ***M****_i_* is the resulting moment in direction *i*, ***H****_i_* is the applied field in direction *j*, and *K_ij_* = *K_ji_*. Because magnetic features, especially carbonate assemblages, were stronger and more numerous in the ARM maps, many of the sources that yielded robust NRM components were no longer well isolated in ARM maps due to spillover magnetic fields from these nearby sources. Anisotropy tensors could accordingly not be confidently calculated for some sources.

To consider a computed anisotropy tensor robust, we required that the fitted source heights for the included ARMs be consistent, with a spread in heights no greater than 50% of the mean sample-sensor height for that source. For sources for which we identified a stable NRM component, we also required that the average fitted height from ARM maps be within 50% of the average source height for the NRM measurements. This criterion is designed to exclude analyses where the fitted ARM signal originated from a different ferromagnetic inclusion than in the NRM analysis or where substantial mixing of magnetic fields from nearby sources is present. If spillover fields from other sources obscured the magnetic field of the source of interest in just one ARM direction, we evaluated variation in fit height and calculated the best-fit anisotropy tensor from the five remaining measurements. AMR tensors were successfully calculated for seven sources, including two for which no NRM component was identified (see section S3).

From these tensors, we computed the corrected degree of anisotropy *P′*

$P^{\prime }={\left(\frac{K_{1}}{K_{3}}\right)}^{\sqrt{1+\frac{T^{2}}{3}}}$ $T=\frac{2\ln(K_{2})-\ln(K_{1})-\ln(K_{3})}{\ln(K_{1})-\ln(K_{3})}$ for each source where *K_i_* represent the ordered eigenvectors of ***K*** (see section S3 and fig. S7). We also computed anisotropy tensors for the sources in 462,9 similarly, using a 340°C TRM imparted along three orthogonal axes. The AMR tensor eigenvectors consistently indicate an oblate fabric with a mean and median *P′*, denoting the corrected degree of anisotropy (*51*), of 2.1 and 2.0, respectively. Because of the apparent consistency of the magnetic fabric, sources for which an independent AMR tensor could not be calculated were corrected using the average anisotropy tensor.

### Paleointensity estimation

We used the ARM normalization method to estimate paleointensities for AF-demagnetized sources and comparison between stepwise thermal NRM demagnetization and pTRM acquisition for thermally demagnetized sources (see section S5). We corrected the single-source paleointensities of sources in slices 462,5 and 462,10 for source anisotropy by multiplying the derived paleointensities by the factor $\frac{\left\|K^{-1}\;\theta_{\mathrm{NRM}}\right\|}{\left\|K^{-1}\;\theta_{\mathrm{ARM}}\right\|}$, where θ_NRM_ and θ_ARM_ represent the fitted NRM and ARM magnetization directions. We used a bootstrapping approach using a database of ARM-TRM correction coefficients to compute means and SEs for each source paleointensity incorporating uncertainty in both fitting and in the conversion of ARM to TRM intensity (*20*). Because experimentally determined correction factors *f′* for pyrrhotite were not available, we estimated values of this factor from *f′* values for magnetite and sIRM to TRM correction factors (*a*) for pyrrhotite given in the database compiled in Weiss and Tikoo (*87*) (see section S5.1).

## Supplementary Materials

### This PDF file includes:

Supplementary Text Figs. S1 to S14 Tables S1 to S6 References
