# Supplementary material for: Paleomagnetic evidence for a long-lived, potentially reversing martian dynamo at ~3.9 Ga
Source: Sci Adv. 2023 May 24;9(21):eade9071. doi: 10.1126/sciadv.ade9071 (PMC10957104; doi:10.1126/sciadv.ade9071)
Supplement: Supplementary file 1 — Supplementary Text Figs. S1 to S14 Tables S1 to S6 References [file sciadv.ade9071_sm.pdf]

Science Advances
AAAS

Supplementary Materials for

Paleomagnetic evidence for a long-lived, potentially reversing martian dynamo at ~3.9 Ga

Sarah C. Steele *et al.*

Corresponding author: Sarah C. Steele, sarah_steele@fas.harvard.edu

Sci. Adv. **9**, eade9071 (2023)
DOI: 10.1126/sciadv.ade9071

This PDF file includes:

Supplementary Text
Figs. S1 to S14
Tables S1 to S6
References

Supplementary Text

1. Fusion crust analysis

To identify and avoid contamination from magnetization acquired during and after Earth atmospheric entry, we performed a baked contact test using seven small segments of fusion crust from samples 462,6, 462,7, and 462,8 and one large segment from slice 462,11 (Fig. S2). We found that the small fusion crust samples displayed moderate scatter around a steep mean NRM direction of D = 315°, I = 64° (Main Text Fig. 4B, gray points). Because substantial VRM has not been reported in the fusion crust of ALH 84001 and the mean fusion crust magnetization direction has been found to be relatively stable up to at least 99 mT (*27*), these small fusion crust samples were not demagnetized and their magnetization directions were calculated from direct NRM measurements. We confirmed this mean direction by thermally demagnetizing a larger sample of fusion crust from sample 462,11 (Fig. S2J) up to 580 °C and measuring its moment with a 2G Enterprises Model 755 superconducting rock magnetometer. Fitting the resulting component, which persisted until 550 °C, yielded a direction of D = 358°, I = 75° with a mean angular deviation (MAD) of 6°. The average direction of these eight measurements was D = 319°, I = 66° with an angular 95% confidence interval of 43°. Sources located ≥1.5 mm away from the fusion crust did not exhibit this direction. Furthermore, this direction is not similar to either of the cluster directions, suggesting the sources considered in this study were not remagnetized during or after Earth atmospheric entry.

2. Magnetic mineralogy

2.1. Identification of sulfides in ALH 84001 chromite

Since chromite in ALH 84001 is expected to be paramagnetic at room-temperature, the strong magnetic moments associated with these grains must have another mineralogical source. Using reflected light microscopy, we identified a number of sulfide inclusions within ALH 84001 chromites. No other potentially ferromagnetic opaque mineral phases were identified. Exposed sulfides are associated with strong signals in QDM maps (Fig. S4).

To determine the compositions of these inclusions, we obtained semi-quantitative energy dispersive spectroscopy (EDS) spectra using a JEOL-7900F scanning electron microscope housed at the Harvard Center for Nanoscale Systems. We collected spectra from 58 locations (2 orthopyroxene, 10 chromite, 46 inclusions within chromite) in slices 462,5 and 462,10 (Table S2, Table S3, Fig. S3). The measurements sampled twenty inclusions across five chromites. All inclusions sampled had compositions consistent with pyrrhotite, pyrite, or a combination of the two within the sample volume (Table S3). This finding agrees with a previous identification of monoclinic pyrrhotite associated with chromite from transmission electron microscopy (TEM) measurements (*27*). No other minerals capable of carrying magnetic remanence have been found in association with the chromite assemblages.

In agreement with this result, chromite-sulfide assemblages in our study were completely demagnetized by 320 - 340 °C as expected for the Néel temperature of pyrrhotite (320 °C) (*88*). Along with the results of EDS composition analysis, this suggests that pyrrhotite is the only room-temperature remanence-carrying phase in ALH 84001 chromite-sulfide assemblages.

Unfortunately, the grains identified here are too small to use classical rock magnetic experiments such as vibrating sample magnetometry (VSM) and alternating gradient magnetometry (AGM) to determine their domain properties. The high observed coercivities may

indicate relatively small grain sizes (*89*), potentially indicative of single-domain or pseudo-single-domain grains.

2.2. A magmatic origin of ALH 84001 chromite-hosted sulfides

Although these sulfides are hosted by a primary igneous phase, this is insufficient to classify them as primary magmatic minerals. These sulfides may instead have been deposited into cracks in the chromite grains during the relatively late carbonate-forming aqueous alteration event. While the euhedral shapes of many sulfides are suggestive of a magmatic origin (e.g. Fig. S5), we collected EDS measurements of platinum group elements in these sulfides to provide independent geochemical constraints on this question.

During fractional crystallization in a magma chamber, platinum group elements (PGEs) partition strongly into sulfides over chromite and silicate minerals (*90*). Magmatic sulfides therefore exhibit characteristic strong PGE enrichments immediately after crystallization. Because PGEs are immobile in a variety of aqueous environments—especially so under the low-T aqueous conditions experienced by ALH 84001 (*91*)—PGE enrichments can potentially be used to identify magmatic sulfides even after periods of known aqueous alteration. If ALH 84001 sulfides crystallized during magmatic cooling, they should retain measurable PGE enrichments. On the other hand, late precipitating sulfides forming in low-temperature fluids should be PGE-poor, with perhaps only trace quantities of the most fluid-mobile PGEs like Pd (*91*). It is also possible for PGE-bearing magmatic sulfides (including those with PGE-containing formulas such as laurite, braggite and cooperite) to recrystallize into other sulfide species (e.g., pyrite, pyrrhotite) *in situ* during fluid alteration while maintaining significant PGE enrichments, especially in the least mobile PGEs like Ru and Pt (*91,92*). Accordingly, while a lack of PGE enrichments would be inconsistent with a magmatic origin, the presence of PGE enrichments does not fully preclude late alteration of the sulfides.

We used measured EDS spectra of ALH 84001 sulfides and chromites to determine their major element and PGE abundances. Sulfides associated with chromite are strongly enriched in Pt and Ru (up to ~0.8 wt% each) relative to both chromite (Fig. S6) and CI chondrites. We did not identify significant enrichments in other PGEs, most notably Pd, but these elements could be enriched at levels below the measurement uncertainty.

Enrichment in Pt and Ru, but not Pd, likely reflects the interaction of ALH 84001 sulfides with water at low temperatures. At 20 °C, Pd is more soluble than Pt and Ru by many orders of magnitude. These solubility differences remain pronounced even at 200 °C (*91*). The PGE enrichment pattern of ALH 84001 sulfides (Ru, Pt rich; Pd poor or absent), coupled with their textural association with magmatic chromites and lack of intergrowth textures (Fig. S4, S5), suggest they most likely originated as magmatic sulfides and later equilibrated with low-temperature fluids, leaching some of their fluid-mobile elements without entirely recrystallizing.

The implied magmatic origin of this pyrrhotite then has several further consequences. First, these sulfides in principle may record magnetic fields as old as 4.091 ± 0.030 Ga, when ALH 84001 initially crystallized. As discussed in the Main Text, the D1 impact event, which may have occurred shortly after crystallization, is effectively the oldest possible age for ALH 84001 magnetization. Second, if the sulfides avoided late major recrystallization, the magnetizations they carry are likely to be thermal instead of chemical in origin and yield robust paleointensities.

Even if sulfides were partially or fully recrystallized, the implications for our overall interpretations are minimal. Since even full recrystallization can occur without unblocking the initial TRM (*55*), a chemical magnetization (CRM) could date either to the D1 event or the

carbonate-forming event. Since the timing of D1 is bounded on the young end by the carbonate-forming event, either scenario would not change our estimate of the magnetization's age, which still corresponds to the age of D1. Although the paleointensity calibration for a CRM would likely differ from typical TRM paleointensity calibration, the true value would likely be within tens of percent of the inferred value but could be either higher or lower (*55*). This implies that whether or not the sulfides recrystallized and/or were remagnetized, we come to the same ultimate interpretation: one magnetization was acquired between 4.0-3.9 Ga, another was acquired at ~3.9-3.8 Ga, and both paleointensities are moderately uncertain but strong enough to likely be of dynamo origin. However, we stress that since sulfides in chromite interiors are petrographically and compositionally indistinguishable from those in cracks or on chromite exteriors, it is unlikely that extensive recrystallization occurred.

Since pyrrhotite is the only known room-temperature remanence-carrying phase in ALH 84001 chromite-hosted sulfides, these assemblages should be entirely remagnetized during impacts with equilibration temperatures above pyrrhotite's Néel temperature. This places strict requirements on the equilibrium temperatures experienced by the ALH84001 host rock during late impact events, including the D3 event, as some sources must have avoided late remagnetization in order to retain records of strong dynamo fields. See Main Text and Supplementary Materials Section 6 for further discussion of this point.

3. Anisotropy of magnetic remanence (AMR)

When a magnetic assemblage more readily acquires remanent magnetization in certain directions, it is said to have anisotropy of magnetic remanence (AMR). Anisotropic magnetic fabrics can arise naturally in some rocks, such as lava flows, but can also be imparted by post-emplacement processes such as sedimentary compaction and metamorphism. Impacts, in particular, can impart oblate magnetic fabrics (*93,94*).

While quantifying the anisotropy of ALH 84001 chromite-sulfide assemblages could potentially provide insight into the meteorite's deformation history, it is also necessary to correctly estimate source directions and paleointensities. If a source was magnetized along any direction other than one of the principal axes of its anisotropy tensor its magnetization direction will be rotated from the true direction of the magnetizing field. The corrected magnetization direction is given by $\theta'_{NRM} = K^{-1}\theta_{NRM}$, where θ_{NRM} is the Cartesian representation of the fitted NRM vector moment and K^{-1} is the inverse of the anisotropy tensor. Additionally, if a laboratory ARM or TRM is imparted in a direction different from the magnetization direction, the resulting paleointensity must be corrected for these anisotropy effects by multiplying the derived paleointensities by the factor $\frac{\|\boldsymbol{K^{-1}}\,\theta_{NRM}\,\|}{\|\boldsymbol{K^{-1}}\,\theta_{ARM}\,\|}$.

We quantify this anisotropy by repeatedly measuring the sample after imparting ARM in known directions then using least squares to compute the tensor *K* that most accurately maps a magnetizing field and direction to a resulting magnetic moment and direction in the sample (*51*). In this study, we used fields imparted along the six directions (*D,I*) = (0,0), (120,0), (240,0), (0,36), (120,36), (240,36) to compute AMR tensors. These represent one orthogonal group of directions and one group of low-inclination directions. Although the QDM fitting methods described here have small errors for the highly dipolar sources we consider (*81*), we chose these laboratory field directions to maintain a constant inclination within each group to mitigate potential biases in fitting high inclination sources. We determined the best-fit anisotropy tensor *K* for each individual source as the least-squares solution of:

$$M_i = K_{ij} H_j \quad (1)$$

where $\boldsymbol{M}_i$ is the resulting moment in direction i, $\boldsymbol{H}_i$ is the applied field in direction j, and $K_{ij} = K_{ji}$. We then determined the shape of anisotropy from the shape parameter T, defined by:

$$T = \frac{2\ln(K_2) - \ln(K_1) - \ln(K_3)}{\ln(K_1) - \ln(K_3)} \quad (2)$$

where K_i represent the ordered eigenvalues of the anisotropy tensor (*51*). In this framework, $0 < T < 1$ defines an oblate (planar) fabric and $-1 < T < 0$ defines a prolate (linear) fabric. We also computed the corrected degree of anisotropy, P':

$$P' = \left(\frac{K_1}{K_3}\right)^{\sqrt{1+\frac{T^2}{3}}} \quad (3)$$

The sources studied here had a mean and median P' of 2.1 and 2.0, respectively, making them moderately anisotropic. Shape parameters for sources in our sample corresponded to a mild to moderately oblate fabric for all but one case, consistent with an impact origin for this magnetic fabric. Values of T for these oblate sources were highly variable, however, with a mean $T = 0.19$ and standard deviation of 0.47. Given the several impacts to which ALH 84001 was exposed, this may reflect different degrees of compaction during distinct impacts or heterogeneous compaction during a single event.

4. Probability of clast rotation as the cause of multiple NRM directions

As an alternative to the hypothesis that the large angle between the two directional clusters represents a reversal record, we considered the possibility of a whole-sample rotation between heterogeneous heating events (Fig. 5 in Main Text, bottom panels). Although estimates of ALH 84001's burial depth range from several meters to several kilometers (*28*, *95*), it most likely resided at a depth of several meters for much of its late history. In the absence of large-scale modification processes such as plate tectonics, impact rotation of large clasts or megaregolith blocks is the most likely candidate process by which ALH 84001 may have been rotated between magnetization events in a field of constant direction. We quantify the likelihood that a random rotation would produce two clusters that are at least as antipodal as the two identified here.

Although small rotations may be more likely, we make the conservative assumption that all final rotation states are equally probable. For two directional clusters separated by 142°, we wish to quantify the probability that a random rotation would be greater than or equal to this angular separation. If all final rotated positions on a sphere are equally likely, the probability that a random rotation will generate an angular separation less antipodal than observed—that is, a separation *less* than the cluster separation angle θ—is equal to the fractional area of the spherical cap subtended by the angle θ. The probability of producing a rotation at least as antipodal as we observe—that is, *greater* than or equal to θ—can then be approximated by the fractional area of the spherical cap defined by the angle $\theta' = 180° - \theta$ (Fig. S8).

This probability approaches 0.11 in the limit where the clusters are very well-defined and separated by exactly 142°. However, because the confidence intervals of the clusters are large, their true center locations are not well known. In this case, geometry dictates that the true cluster centers are more likely to lie at a smaller angular separation that the 142° reported and the approximation above breaks down. A better approximation of this probability can be obtained using a parametric bootstrap wherein we sample possible true cluster centers repeatedly from the Fisher distributions defined by each of our clusters, compute the angular separation of each

resampled pair and the corresponding probability that a random rotation would exceed that angle, and analyze the resulting distribution.

To this end, we began by drawing 5000 samples from each of the Fisher distributions defined by the means and α_{95} values of the two directional clusters. We then calculated the angular separation θ for *all combinations* of resampled cluster centers (Fig. S9A). Computing the probability that a random rotation would exceed θ for each pair of resampled cluster centers yielded a broad, skewed probability distribution with a median of 0.22 (Fig. S9B). Physically, this represents the typical probability that a random rotation would result in more antipodal directions than our dataset given the range of true separations between the observed clusters due to their uncertainty.

Since we made the conservative assumption that all final rotated positions are equally likely, this probability estimate is an upper bound. It is therefore unlikely that the large angular separation between the two directional clusters was produced by clast rotation between heterogeneous heating events.

5. Paleointensities

5.1. Paleointensity estimation and uncertainty quantification

When a room temperature paleointensity protocol is needed for alteration-prone samples, AF demagnetization and ARM acquisition can be sufficient analogues for thermal demagnetization and acquisition, respectively (*47, 96*). However, ARM paleointensities require correction for the differing efficiencies with which ARM and TRM are acquired in a given field. The true paleointensity B_{PI} can be estimated from

$$B_{PI} = \frac{\Delta NRM}{\Delta ARM} \cdot \frac{B_{bias}}{f'}$$

where B_{bias} is the bias field applied during ARM acquisition, ΔNRM and ΔARM are the respective changes in NRM and ARM over a given AF range, and f' is the correction factor. The value of f' is challenging to estimate because it depends on the magnetic mineralogy and domain state, which are generally underconstrained.

We followed the approach of Volk et al. (*20*), employing a bootstrap method of estimating paleointensities to account for uncertainties in both the fitting and the appropriate value of f'. Our methods differed from those of Volk et al. (*20*) in the selection of a pool of f' values since the magnetic mineralogy in their study was dominated by magnetite, for which robust estimates of f' exist over a broad range of grain sizes and domain states (*87*). For pyrrhotite-dominated mineralogies considered in this work f' is not well characterized.

Weiss & Tikoo (*87*) performed a literature review of f' and a (the calibration coefficient for saturation isothermal remanent magnetization (sIRM)-normalized paleointensities). While the library they assembled lacks f' data for pyrrhotite, they include a data set for a that samples a variety of pyrrhotite mineralogies and domain states. Since the mineralogy and domain state of pyrrhotite in our sample remain poorly constrained, we used this entire data set to generate estimates of f'.

The shapes of the distributions of f' and a for magnetite are similar (Fig. S12). We therefore assume that f' for a given mineralogy is related to a for that mineralogy by a simple multiplicative scaling. To account for the different sizes of the two data sets, we mapped each f' value for magnetite to the a value that occupied the same percentile location in the distribution and computed the ratio between the two. This yielded a fairly narrow distribution with $\overline{f'/a}$ = 0.0012 ± 0.0002.

Assuming the ratio f'/a for pyrrhotite is similar to that of magnetite, we multiplied the available a values for pyrrhotite by the computed mean f'/a value to obtain estimated f' values for pyrrhotite (Table S5).

For each source, we drew 2000 sample ARM paleointensities from a normal distribution defined by the mean and uncertainty in the fitted slope $B_{PI,ARM} = \frac{\Delta NRM}{\Delta ARM} \cdot B_{bias}$. We then paired each of these samples with an f' chosen at random from our set of f' estimates to calculate an estimate of B_{PI}. This process yielded the individual paleointensity estimates in Table S4. The average estimated paleointensities for the two directional clusters are 42 ± 20 μT and 15 ± 11 μT, where the uncertainty values represent the standard errors computed from the bootstrapped distributions for each cluster.

5.2. Weakly magnetized sources

In addition to the two strongly magnetized populations we also identified a population of sources that lacked measurable NRM but were capable of acquiring strong ARM even at very low bias fields. Since the NRM of these sources was weak compared to the magnetic background, dipolarity values for inversions of these sources were often too low to guarantee an acceptable fit. We therefore could not estimate their paleointensities using traditional inversion-based methods.

However, we can take advantage of this property to place an upper bound on the magnetizing field by identifying the smallest ARM bias field that can still be robustly fitted. Because this laboratory magnetization is stronger than the NRM, the TRM equivalent of this ARM bias field can then be interpreted as an upper bound on the ancient magnetic field during its most recent magnetization event. For each identified weak source (e.g. Fig. S13), we applied ARMs with bias fields from 10-200 μT. We found that ARM bias fields above 10-20 μT produced magnetizations that were strong enough to be robustly fitted, suggesting TRM paleointensities could be no stronger than ~2-7 μT. Applying the same bootstrap method detailed above assuming errors of 5 μT in the recording limit due to the spacing of our applied bias fields yields a paleointensity limit of ≤ 3.4 ± 2.8 μT, or <6 μT, for this population.

6. ALH 84001 thermal history

A number of possible thermal histories have been proposed for ALH 84001 since its identification as a martian meteorite. The first consolidated history of ALH 84001, presented in Treiman (*97*) and updated in Treiman (*24*), established the D1, D2, etc. naming convention for major events in ALH 84001's thermal history. We adopt this convention in this work. In light of the many additional constraints placed on the thermal history of ALH 84001 in recent years, we propose here an updated thermal history for ALH 84001, including summaries of major thermal events, key constraints on their magnitudes and timings, and their paleomagnetic implications. A summary of these constraints is available in Table S6.

Igneous crystallization. Although the igneous crystallization age of ALH 84001 was initially placed at ~4.5 Ga based on Rb-Sr and Sr-Nd ages (*17*), Lapen et al. (*21*) recognized that these ages may reflect mixing or chemical alteration of ALH 84001 phosphates. The most robust estimate of ALH 84001's crystallization age is currently 4.091 ± 0.031 Ga based on Lu-Hf and Pb-Pb ages (*18*, *21*). The true crystallization age of ALH 84001 may be earlier if these chronometers date a metamorphic event other than post-igneous cooling (*38*).

D1. This event—likely a large impact—was responsible for producing the granular bands and chromite stringers in ALH 84001 and homogenizing the rock's composition. It may correspond to either the 4.163 ± 0.035 Ga $^{40}Ar/^{39}Ar$ isochron age from Cassata et al. (*37*) or any of the 4.0-4.1 ages from other studies (*17*, *25*, *98, 99*).

Substantial metamorphism occurred nearly contemporaneously with, and may be related to, the D1 event. Heating during this metamorphism was sufficient to form 120° triple junctions in some silicate phases; a temperature of 875 °C has been estimated from Ca-Mg-Fe exchange of adjacent contemporaneous orthopyroxene and augite (*97*). Any existing paleomagnetic record in the chromite-sulfide assemblages would have been completely overprinted during this heating. D1 is therefore the earliest possible age of any preserved remanent magnetization in ALH 84001.

Carbonate formation. Carbonates precipitated along fractures into void spaces (or glasses, if earlier generations of glass existed) at 3.95 ± 0.02 Ga based on Rb-Sr and Pb-Pb ages (*29*, *30*). The temperature and general conditions of this event are uncertain, although recent estimates suggest carbonates formed in a relatively cool (18 ± 4 °C) flood evaporitic setting (*28*). If surface temperatures on Mars were similar to those at present, carbonate deposition at even this low temperature would require moderate heating by an impact or other hydrothermal source. However, such a heating event alone would not likely be of any paleomagnetic significance.

D2. While "D2" in Treiman (*24*) refers to an event that predates carbonate formation, based on cross-cutting relationships and the replacement of some plagioclase glass by carbonate, the revised history in Treiman (*38*) excludes this pre-carbonate formation event. This change reflects the more recent recognition that deformations initially attributed to two separate impacts—one before and one after carbonate formation—may be accomplished in just one (*100*). Accordingly, this work uses "D2" to refer to an event that postdates carbonate formation.

This event aligns with the $^{40}Ar/^{39}Ar$ plateau at ~3.9 Ga identified in several studies (*36*, *99*) and possibly with a 3.84 ± 0.05 Ga Rb-Sr age (*101*). Local temperatures during this event were high enough to generate and mobilize plagioclase melt, though the limited melting of other phases (e.g. carbonate, orthopyroxene) suggests temperatures this high could not have been homogeneous. The equilibrium temperature of this event is therefore uncertain. Since many regions of the rock retain older $^{40}Ar/^{39}Ar$ ages, sustained temperatures during this event were likely not high enough to fully reset this chronometer. Our paleomagnetic results, which indicate two high-field magnetizations were preserved, also support equilibrium temperatures below 320 °C for this event. Modeling of impact heating for the 30-45 GPa pressures inferred for this event affirms that equilibrium temperatures could not have been higher than 320 °C (*41*).

D3. Although this event left no unambiguous petrographic signatures it is associated with a $^{40}Ar/^{39}Ar$ plateau at 1.158 ± 0.11 Ga (*37*) and potentially also a 1.39 ± 0.10 Ga Rb-Sr age (*101*). This event has potentially critical implications for the paleomagnetic interpretation since peak temperatures >1400 °C have been proposed during this impact. A heating event of this magnitude, which would imply pyrrhotite was completely remagnetized after the dynamo's cessation, appears inconsistent with the paleomagnetic results from our work and others showing multiple sets of magnetization directions. It is also unclear how the impact properties required to produce such high temperatures could be reconciled with the estimates of impact pressure from plagioclase glass in the sample (*40*).

Heterogeneous impact heating may explain this discrepancy. A brief, 1400 °C event was conceived by Cassata et al. (*37*) to account for the observation that, in certain aliquots of ALH 84001, orthopyroxene experienced more Ar degassing than maskelynite. The latter mineral is expected to degas more efficiently than orthopyroxene at low temperatures but is more retentive above ~1400 °C. As such, the authors proposed that the bulk rock must have been heated to above that temperature. Alternatively, orthopyroxene may have been preferentially degassed if it was heated to systematically higher temperatures than maskelynite. Modeling of heterogeneous impact heating in ALH 84001 demonstrated that orthopyroxene could be systematically heated to 100-200 °C higher than plagioclase across a range of impacts (*41*). These mesoscale impact simulations confirmed than a 45 GPa impact could reproduce this excess degassing of orthopyroxene with substantially lower peak temperatures in orthopyroxene and maskelynite of 1050 °C and 900 °C, respectively. Since the equilibrium temperature of such an event would have been ~300 °C many chromite-sulfide assemblages, including our thermally demagnetized sources 9a, 9b, and 9f, would have avoided total remagnetization. As well as providing a mechanism to create the unique magnetization of ALH 84001 chromites, heterogeneous impact heating may then reconcile the $^{40}Ar/^{39}Ar$ data from Cassata et al. (*37*) with paleomagnetic results showing non-unidirectional magnetization components (this work and refs. *22, 27, 42, 43*).

A lower temperature for this event may also align more closely with other $^{40}Ar/^{39}Ar$ data. First, the young $^{40}Ar/^{39}Ar$ ages by which this event was identified were only found in a fraction of samples, and only in a single study (*37*). Second, constraints from phosphate (U–Th)/He and whole-rock $^{40}Ar/^{39}Ar$ ages suggest this event could have heated the rock to no more than several hundred degrees for less than a few hours (*102*). These observations suggest high temperatures were not uniform and require low enough peak temperatures in maskelynite and orthopyroxene to accommodate low equilibrium temperatures in the bulk rock.

The overall thermal picture of the D3 event suggests chromite-sulfide assemblages in ALH 84001 would not have been uniformly remagnetized. Based on $^{40}Ar/^{39}Ar$ data, Cassata et al. (*37*) suggested appropriate strong upper bounds of 80 °C for prolonged cooling (10 Ma) or 330 °C for brief cooling (several days). However, since $^{40}Ar/^{39}Ar$ dating constrains the integrated heating, transient localized high temperatures immediately post-impact (e.g. North et al. (*41*)), other lower-temperature impact events, or higher ambient temperatures would all lower the permitted equilibrium temperature for this event. It is therefore likely that magnetizations of many ALH 84001 chromite-sulfide assemblages survived this impact.

D4. The last high-temperatures event in the history of ALH 84001 was its ejection from Mars at 15 Ma (*107*). Early modeling suggested that equilibrium temperatures during this event could have been no higher than 400 °C if cooling occurred in <1 hour (*102*). Cassata et al. (*37*) argued that more realistic strong upper bounds ranged from 75 °C if the rock cooled over 10 Ma to 320 °C for cooling over several days. Again, the true equilibrium temperatures of these events were likely much lower, suggesting remagnetization of chromite-sulfide assemblages was minimal.

Post-D4. The final thermal event in the history of ALH 84001 is its arrival on Earth about 13,000 years ago (*103*). This event is not associated with substantial heating or remagnetization outside of the ~1 mm baked zone (this work and refs. *22, 23*) and the bulk rock was likely not heated above 40 °C during Earth atmospheric entry (*23*).

Other impacts. The timeline given here defines the minimum required set of events and is likely not exhaustive. However, phosphate (U–Th)/He and whole-rock $^{40}Ar/^{39}Ar$ ages impose strict limits on the integrated heating of ALH 84001 after the D2 event, so any late heating events must have been very short-lived and relatively cool. On the other hand, complex crosscutting relationships and scattered $^{40}Ar/^{39}Ar$ ages may mask the signatures of unique shock events prior to 3.9 Ga.

The most importantt uncertainty is whether the deformations attributed here to a single D2 event represent signatures of one or multiple impacts. For example, if carbonate formation occurred in a cool hydrothermal environment, two impacts may be required: one to initiate hydrothermal activity and another to form plagioclase and orthopyroxene glasses, initiate siderite decomposition in the carbonates, and close the void spaces into which carbonates previously precipitated.

Paleomagnetic results may also help constrain the heating history of ALH 84001. Our results suggest an impact must have occurred after the dynamo's cessation to produce the weakly magnetized sources. This is consistent with the randomly oriented magnetizations of the carbonates, which experience substantial impact heating (*41*) and are therefore more prone to remagnetization during late impact events. Unfortunately, our results are not sensitive to the number of small late impacts.

The identification of weakly magnetized chromite-sulfide assemblages also implies that crustal fields in the vicinity of ALH 84001 were too small to be responsible for the strong magnetizations we observe. The two recognized magnetization clusters must therefore record dynamo fields unless the regional crustal magnetic field was also modified between partial remagnetization events. Since local temperatures in the D2 event were high enough to remagnetize some or all chromite-sulfide assemblages, and D3 and the ejection are the only recognized subsequent heating events, at least one of these directional clusters must reflect the field during or after the D3 impact to produce the three total observed magnetization populations. The other likely records the field during either the D1 or D2 event. Better constraints on the number and timing of early impacts may allow paleomagnetic results to place stricter constraints on the martian dynamo.

7. Martian dynamo nondipolarity and reversal asymmetry

The dipolarity of the martian dynamo magnetic field, as approximately quantified by the strength of the dipolar component (G1) relative to the quadrupole (G2) and octupole (G3) components, is not constrained by existing observations. This is important for this work because larger relative G2 and G3 components generate more reversal asymmetry, possibly contributing to or producing the deviation from antipodality we observe in our two directional clusters.

We find that, given the uncertainties in our cluster means, our results are compatible with a purely dipolar field or up to ~3:1 ratios of G3:G1 and G2:G1 (Fig. S14) Even higher G3:G1 and G2:G3 ratios cannot be ruled out at 2σ but are substantially less likely. Because our results admit such a broad range of potential field geometries, they do not appreciably constrain the dynamo's structure besides ruling out a highly multipolar dynamo.

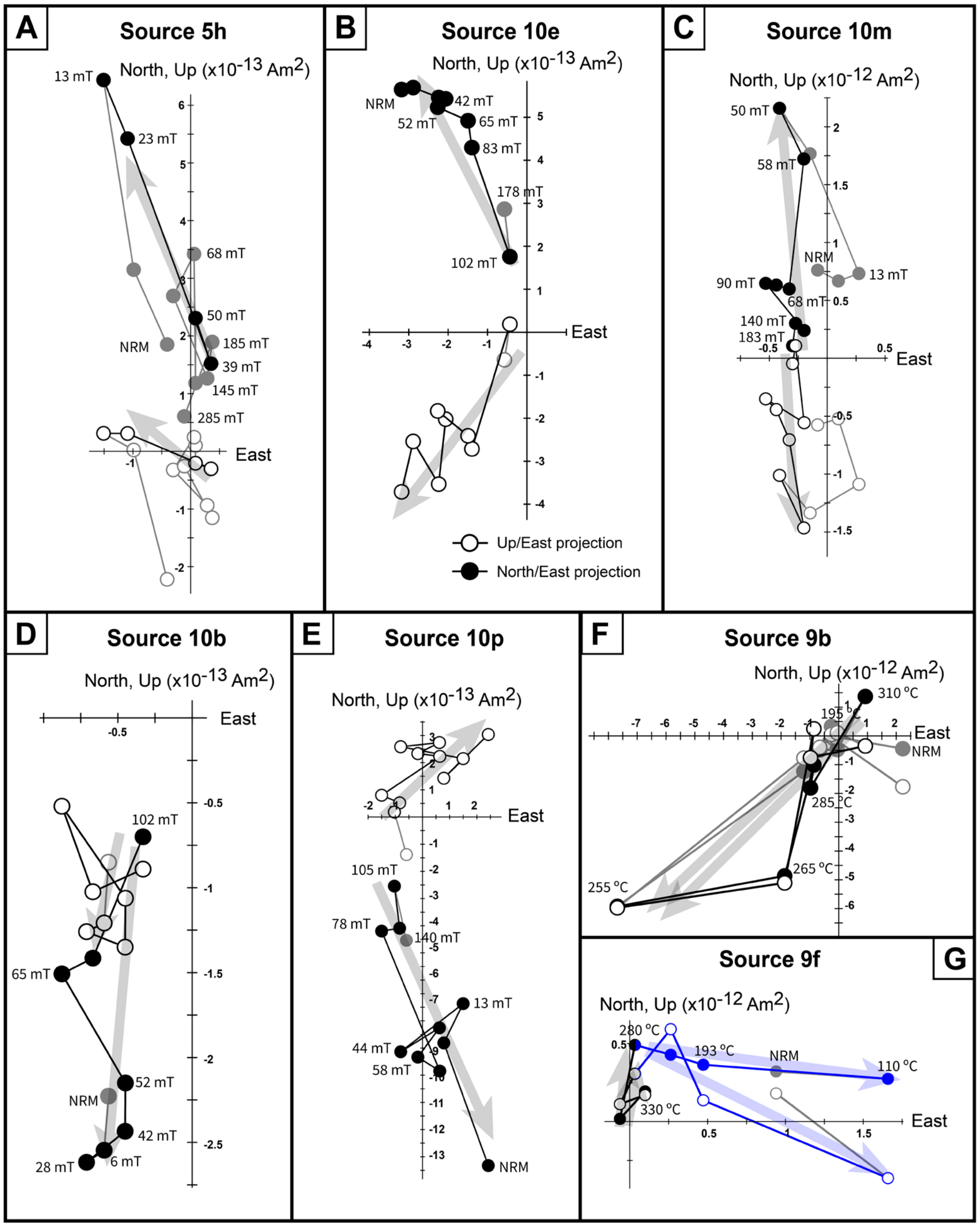

Fig. S1.

Orthogonal projection, or Zijderveld, diagrams for all sources with fitted components not shown in the Main Text. Open and closed points in these panels represent projections onto the Up-East and North-East planes, respectively. The highest coercivity component for each source is shown in black. Low temperature components are shown in blue. Gray points are not included in any component. Plotted steps typically represent averages of multiple demagnetization steps to suppress noise. All sources were demagnetized to 300 mT/ 340 °C, but we exclude steps after sources lose directional coherence and begin to produce unreliable and chaotic fit results. Full demagnetization data for all sources is available in the public data archive (*104*). (**A-E**) AF demagnetized sources. (**F, G**) Thermally demagnetized sources.

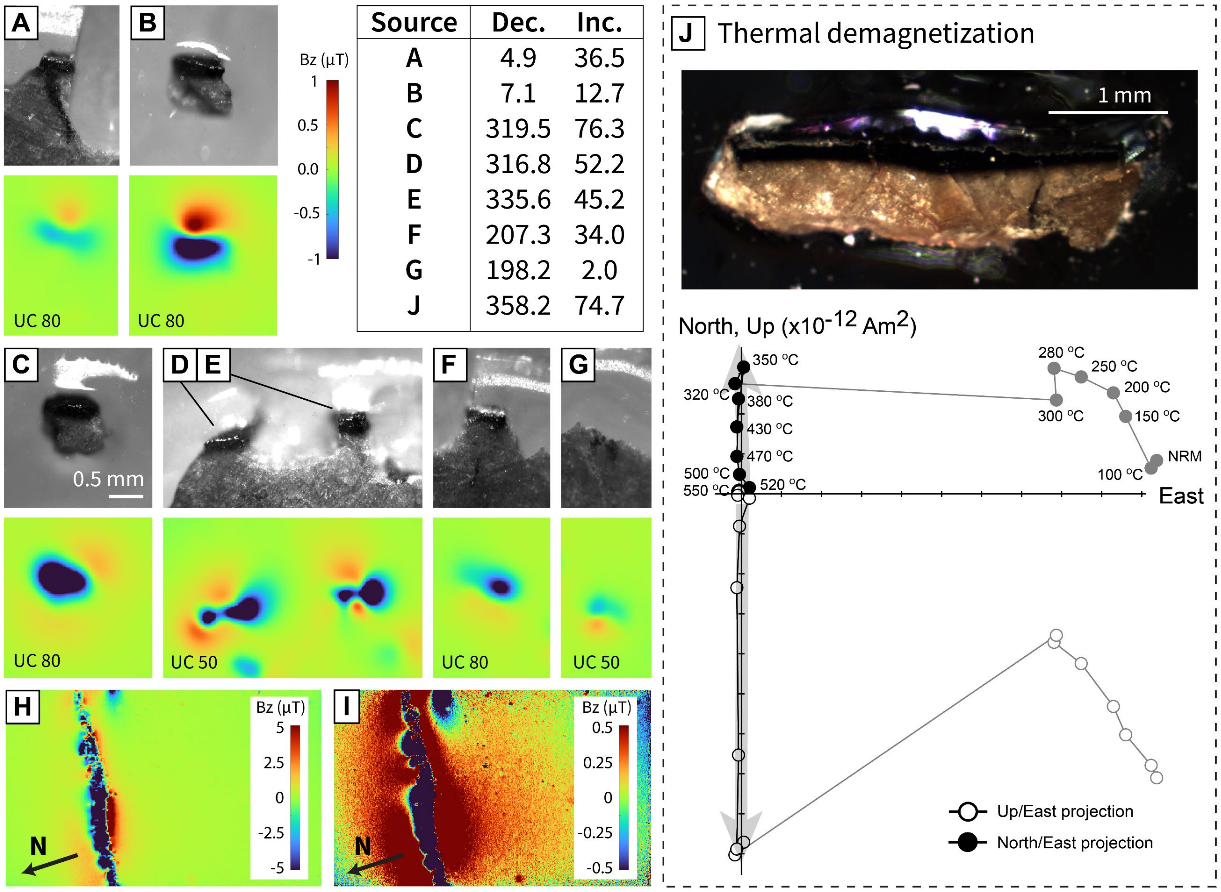

Fig. S2.

Summary of fusion crust baked contact test data. (**A-G**) Visible light images and magnetic field maps of selected fusion crust segments. Green regions are areas of low magnetic field, while red and blue regions have strong negative (into the page) and positive (out of the page) magnetic fields, respectively. Surrounding material was milled out using a non-magnetic dental tool to isolate the magnetic signal of the fusion crust. Fitted directions are shown at right. (**H, I**) Magnetic maps of a continuous section of fusion crust plotted at two saturation levels showing the steep, into-the-page magnetization of the fusion crust. (**J**) A visible light image (top) and thermal demagnetization orthogonal projection diagram (bottom) for a large piece of fusion crust from slice 462,11. The fitted HT component, shown with the gray arrows, is steep and similar to the mean fusion crust NRM direction fitted from smaller fusion crust samples (Fig 4B, gray square).

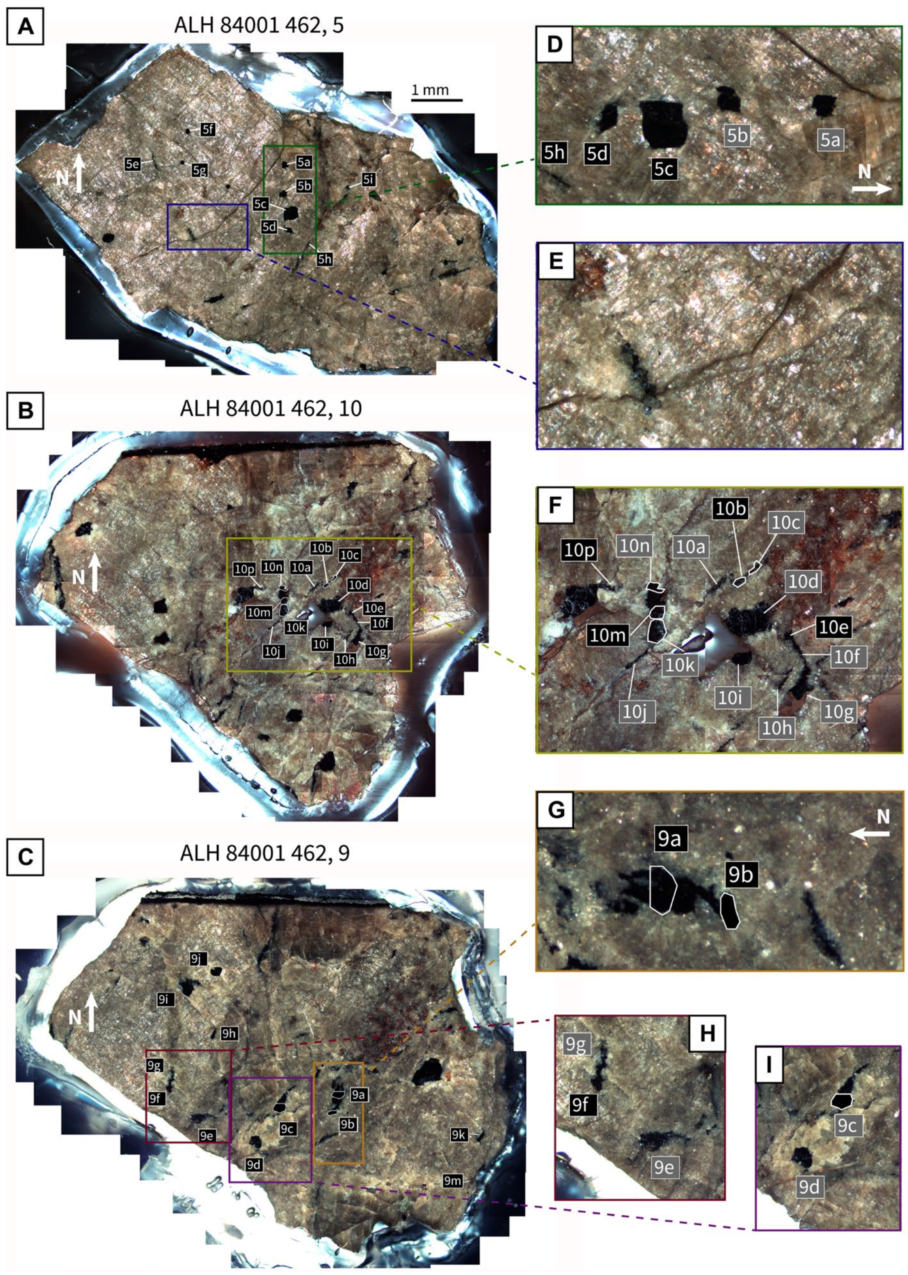

Fig. S3.

Images of the three slices of ALH84001,462 studied in this work. The slices 462,5 (**A**) and 462,10 (**B**) were subjected to AF demagnetization and ARM acquisition experiments, and the slice 462,9 (**C**) was subjected to thermal demagnetization and pTRM acquisition experiments. (**D-I**) Detailed views of the chromite-sulfide assemblages studied in this work and other features of interest. Chromite-sulfide assemblages from which robust NRM demagnetization components were obtained are shown with black labels. In panel E, note a chromite stringer crossing a prominent crack in the sample, suggesting minimal motion has occurred along that crack since the D1 event.

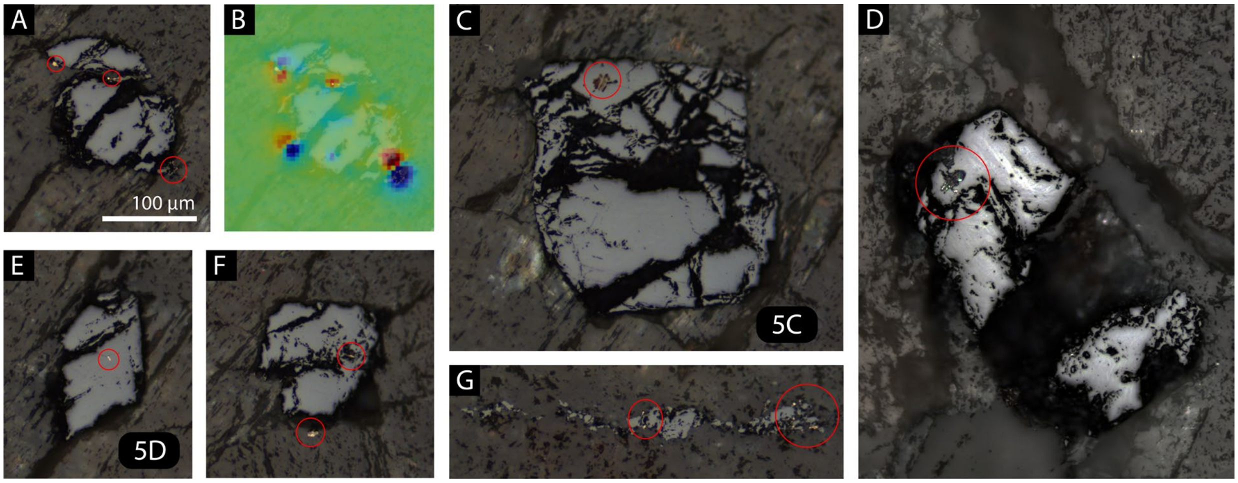

Fig. S4.

Distribution of pyrrhotite within a chromite-sulfide assemblage. (A) A reflected-light microscope image of an ALH 84001 chromite with visible inclusions marked with red circles. (B) A magnetic map of the same chromite overlaid on the microscope image. Note that strong magnetic signals are associated with many of the observed inclusions, which were identified as pyrrhotite during SEM imaging. Magnetic signals that are not clearly associated with visible inclusions likely represent additional sulfides that are not exposed on the surface of the slice. (C-G) Reflected-light microscope images of other typical chromite-sulfide assemblages ALH 84001,462 with visible sulfides marked with red circles. Assemblages studied in this work are labeled. Note that sulfides are distributed in and around chromite, with some sulfides in cracks or on edges and others entirely encased by chromite.

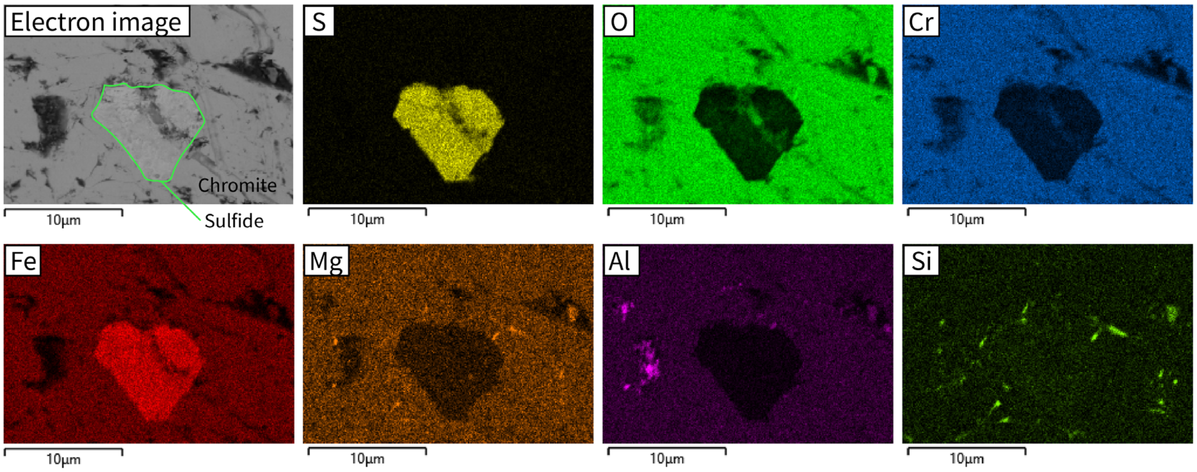

Fig. S5.

EDS compositional maps of a euhedral pyrite inclusion in magmatic chromite. A scratch in the grain's surface of chromite-like composition likely reflects collection of crushed chromite material in the scratch. This sulfide is euhedral, supporting a magmatic origin. It is also located in the interior of a chromite away from any cracks, making it less susceptible to alteration.

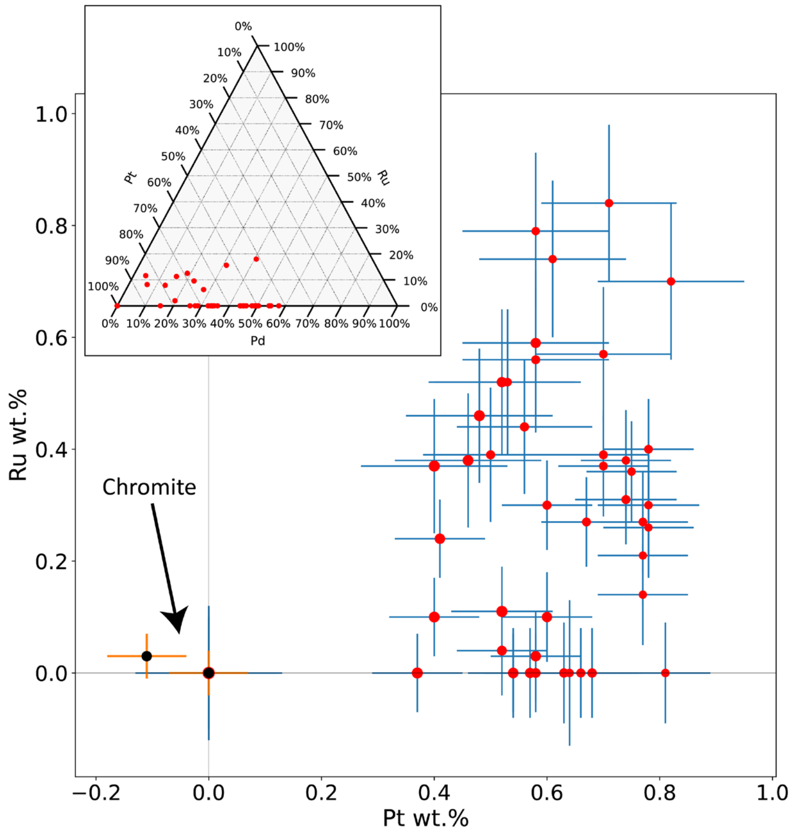

Fig. S6.

Trace element composition of chromites and sulfides. Ru vs. Pt abundances in chromite (black points with orange error bars) and chromite-hosted pyrrhotite and pyrite (red points with blue error bars). For sulfides, the size of data point scales with Fe:S ratio. The inset shows the relative wt. % of Pt, Ru, and Pd in the sulfides.

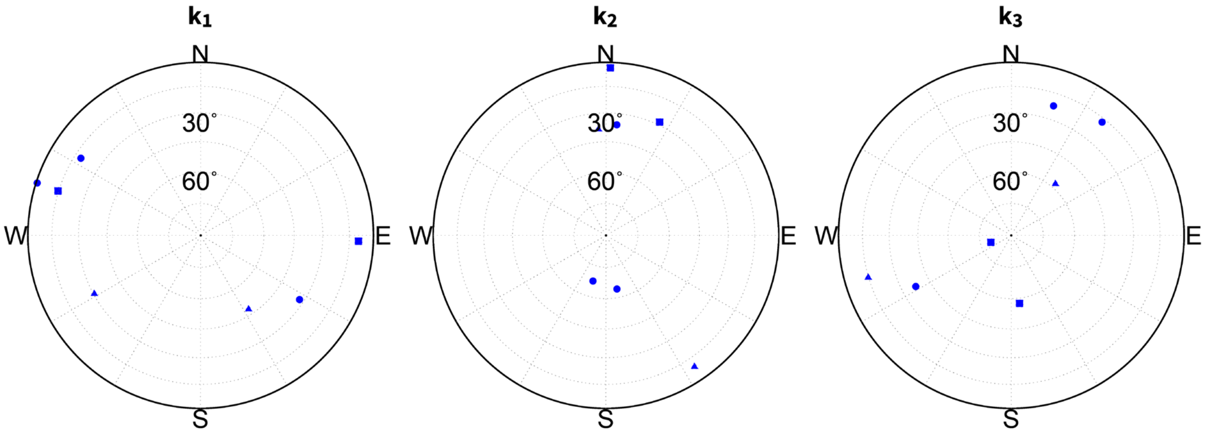

Fig. S7.

Lower hemisphere projections of the easy (k1), intermediate (k2), and hard (k3) eigenvectors of individual source anisotropy tensors. Triangles, squares, and circles represent sources in sample section 462,5; 462,9; and 462,10; respectively.

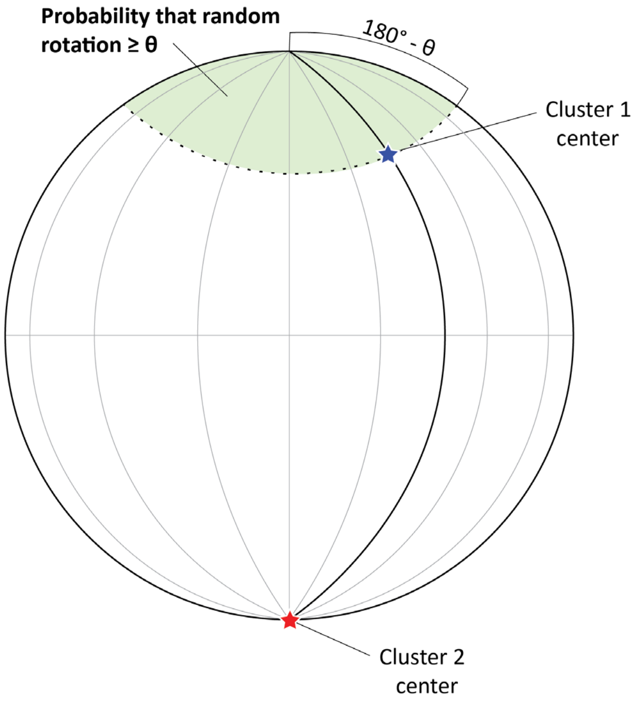

Fig. S8.

Diagram demonstrating the geometry by which we calculated the probability that a random rotation would yield an angular separation ≥ θ for the two clusters. For clusters with uncertainty, where the center is not exactly known, this uncertainty can be propagated by repeating this fractional spherical cap area calculation for a large set of resampled cluster centers. The resulting distribution of probabilities can then be used to estimate an upper-bound probability that the measured angular separation of θ reflects a random rotation.

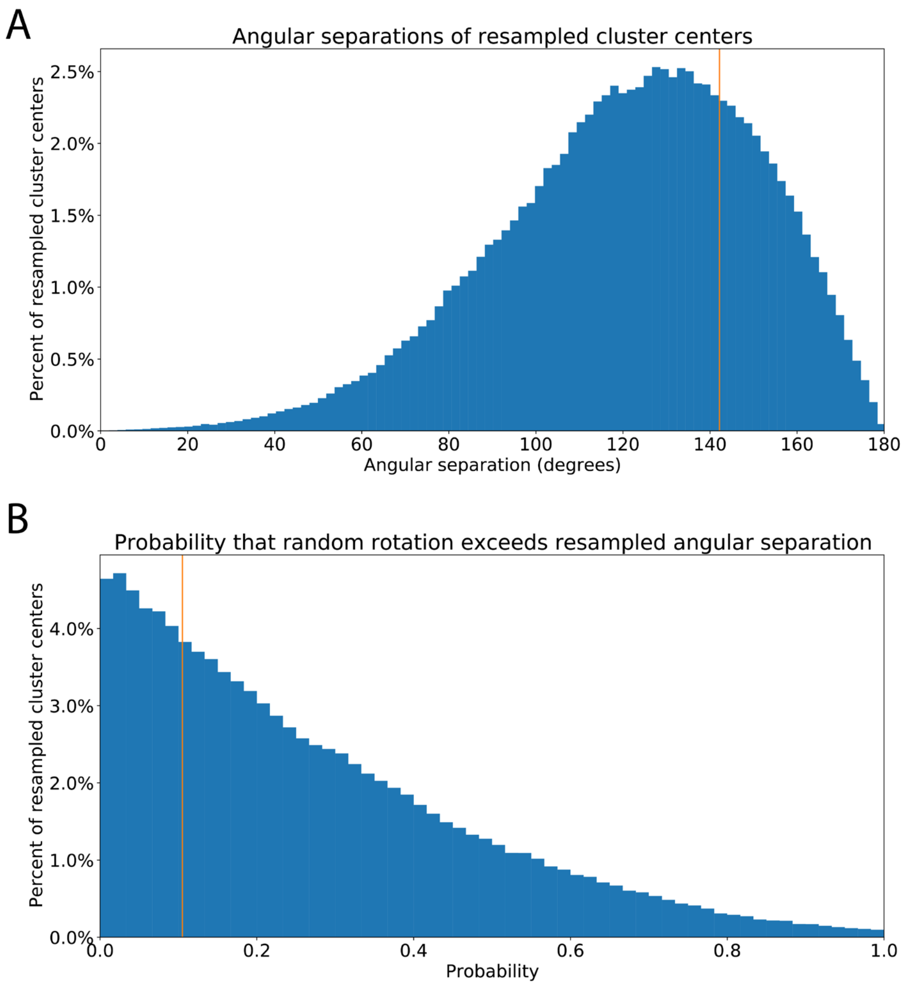

Fig. S9.

Probability analysis for large random rotations. (**A**) Histogram of the angular separations between pairs of resampled cluster centers. The orange line denotes the angular separation of the original clusters (142°). (**B**) Histogram of the probabilities that a random rotation would exceed the angular separations between pairs of resampled cluster centers. In other words, the probability value for a single resampling is the fractional area of the spherical cap outside the unit sphere area between the initial and final directions (Fig. S8). The orange line denotes the probability for well-defined cluster centers separated by 142° (0.11). This distribution is clearly skewed towards lower fractional spherical cap areas stemming from uncertainty in the true centers of the two clusters, and the median fractional cap area in this distribution (0.22) places an upper bound of on the probability that a randomly rotation would make clusters as or more antipodal than our dataset. See Section S4 for further explanation of the quantities plotted here.

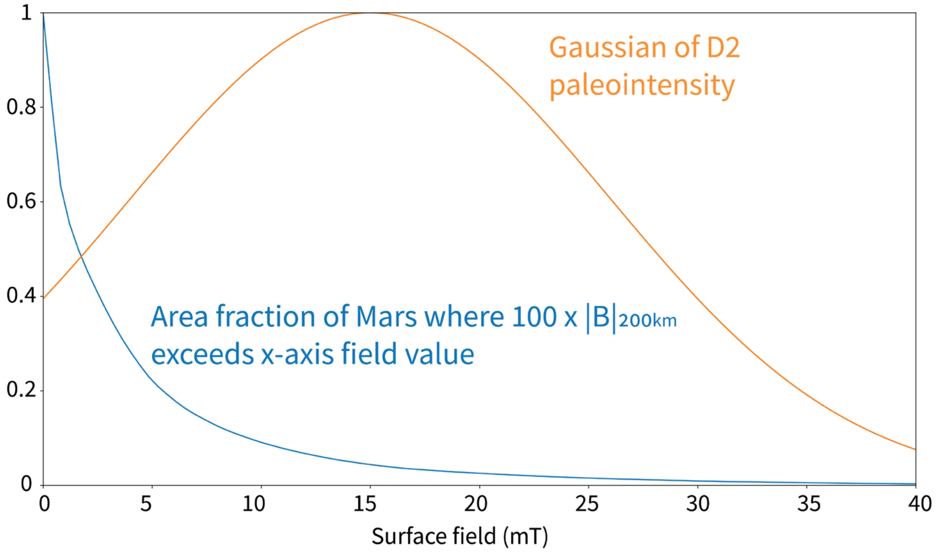

Fig. S10.

Probability that the 15 ± 11 µT cluster paleointensity could be produced by crustal fields. Although only 4% of Mars's surface hosts crustal fields stronger than 15 µT, the considerable uncertainty of our paleointensity estimate means that it may be more plausible for this cluster to have been magnetized by crustal fields. This probability is equivalent to the convolution of the Gaussian representing the cluster's paleointensity (orange) and the fraction of Mars's area where crustal fields exceed a given paleointensity (blue). The latter of these was computed by assuming surface fields are 100 times stronger than those modeled at 200 km altitude (*67*), consistent with surface fields measured by InSight (*66*). From this, we compute a 9% probability that ALH 84001 originated from a region with crustal fields strong enough to produce the 15 ± 11 µT paleointensity of the second cluster. However, since we observe an even more weakly magnetic population in the meteorite, we consider it even less likely that either cluster was magnetized by crustal fields.

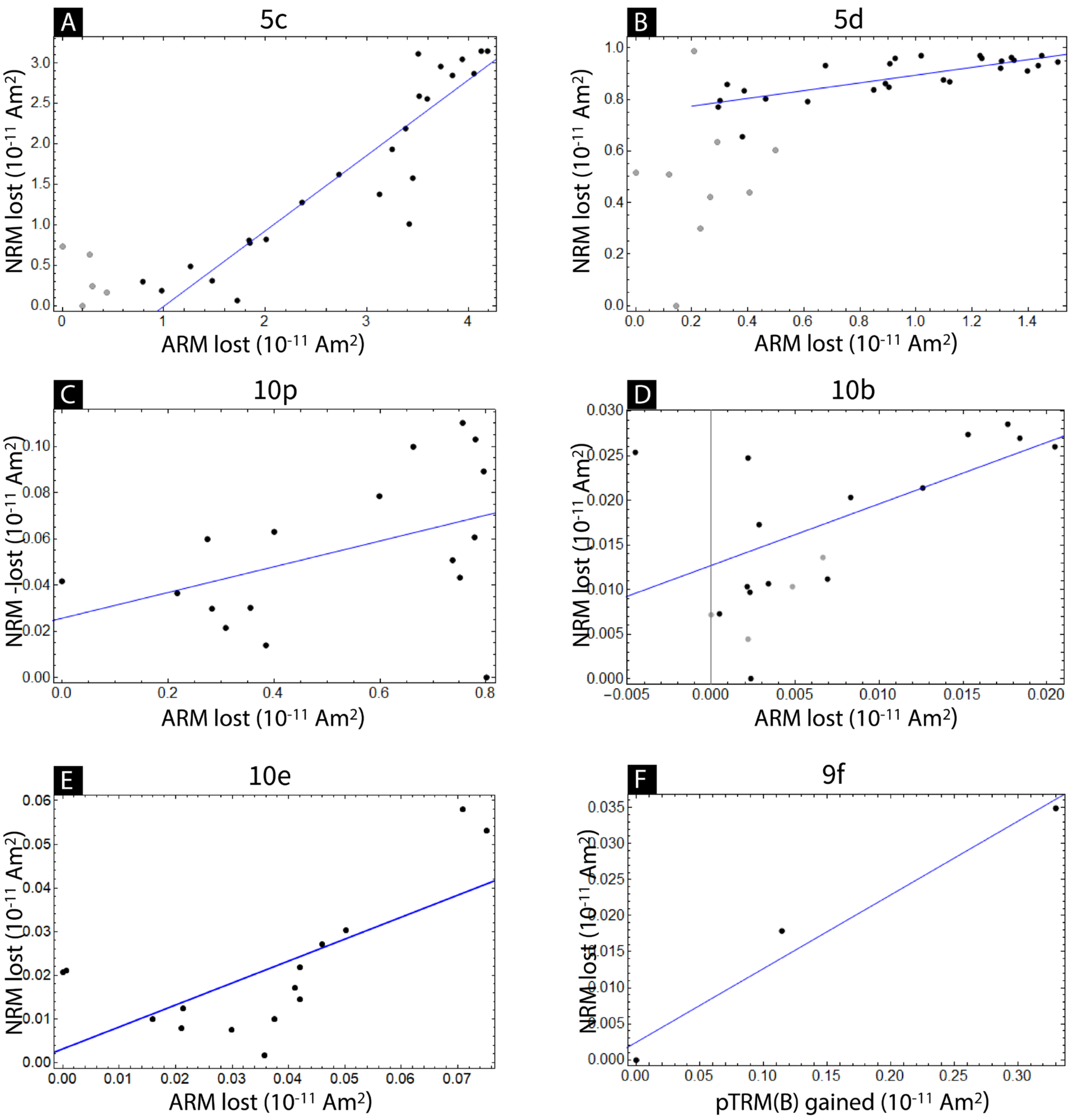

Fig. S11.

Data and least-squares fits (blue line) for ARM (A-E) and pTRM (F) paleointensities. The x- and y-axes represent the total change in ARM (or TRM) and NRM moments with progressive demagnetization, respectively. Points included in the fitted continuous component are shown in black, while points representing demagnetization steps above or below the HC/HT component fit range are shown in gray.

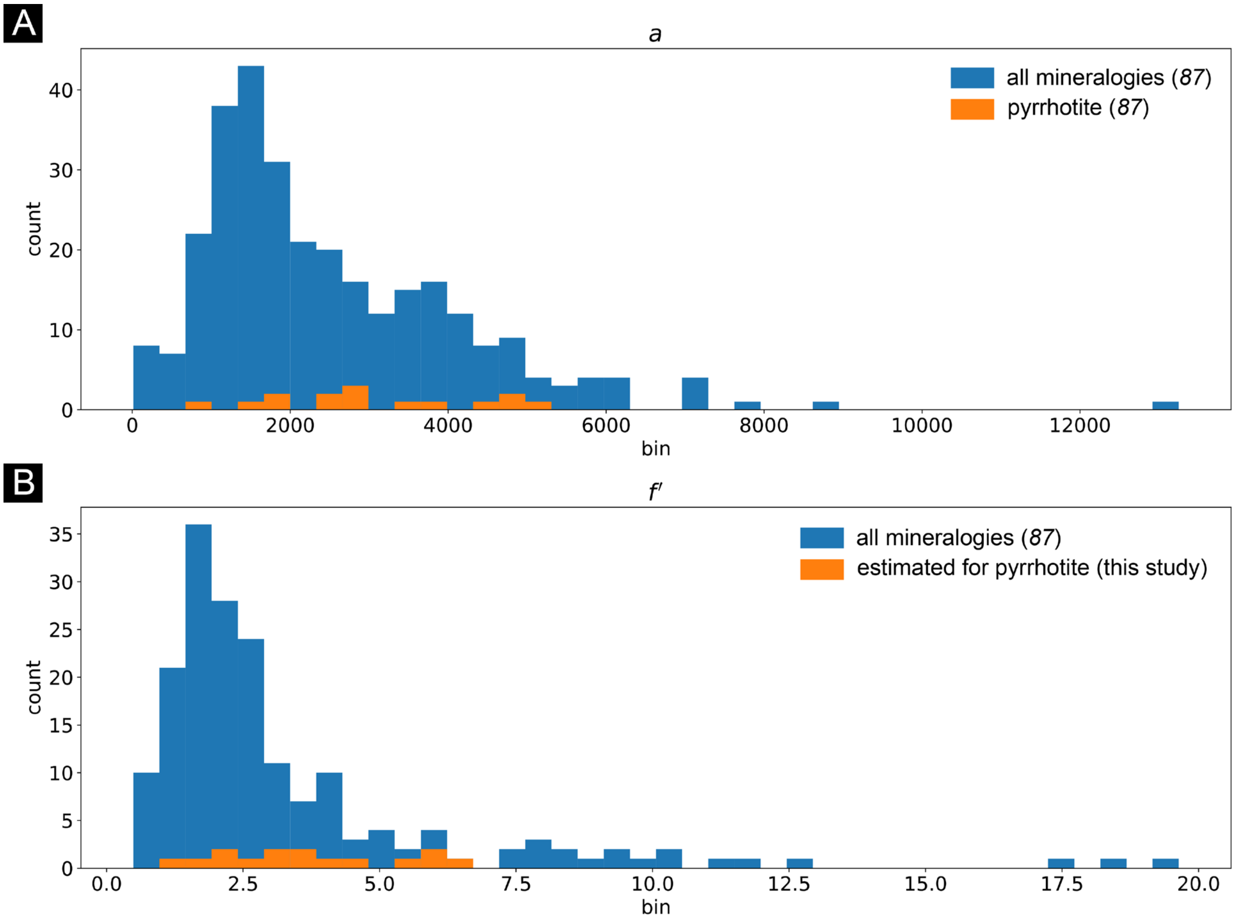

Fig. S12.

Estimation of the TRM to ARM efficiency ratio. (**A**) A histogram of the empirical coefficient describing the ratio of a TRM to an IRM (a) for all mineralogies (blue) and just pyrrhotite (orange) collected by Weiss & Tikoo (*87*). (**B**) A histogram of efficiency ratios between TRM and ARM (f') for all available mineralogies collected by Weiss & Tikoo (*87*) (blue) and estimated for pyrrhotite in this study (orange).

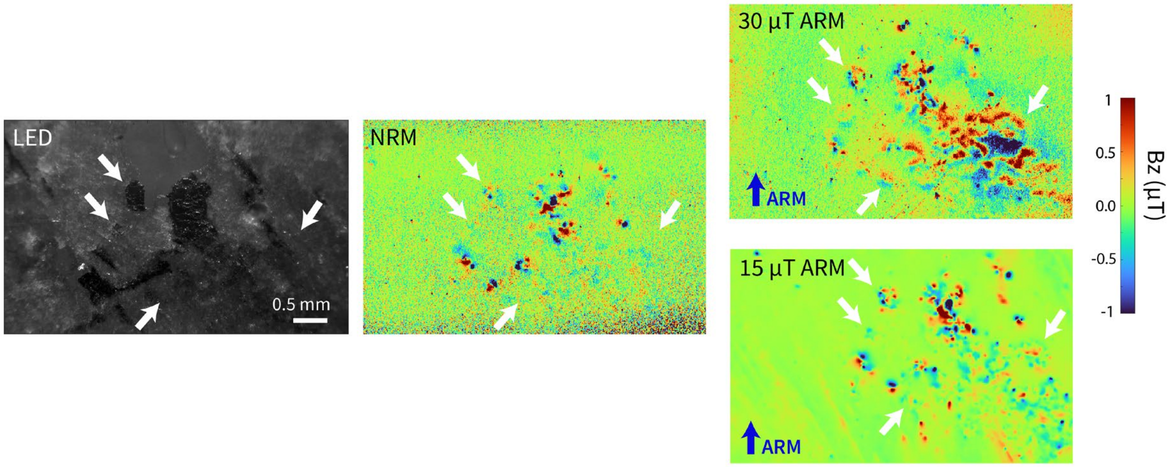

Fig. S13.

A comparison of the NRM (center) and two low-bias-field ARMs (right) applied to the field of view at left. A 30 μT ARM produced uniform magnetization much stronger than the NRM for several chromite-sulfide assemblages and carbonates, denoted in these images by white arrows. A 15 μT ARM produced less coherent, but typically still stronger, magnetization for the same sources. ARM bias fields of 10-20 μT (equivalent to a TRM paleointensity bound of < 6 μT) were required to reproduce the weak NRM in this population.

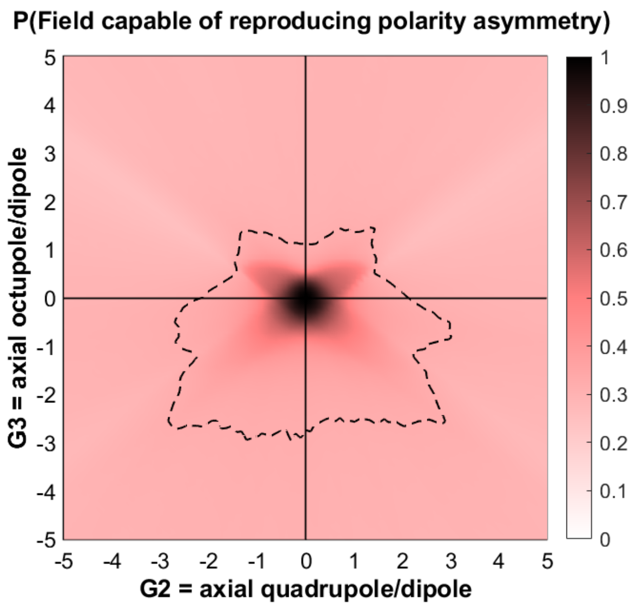

Fig. S14.

Probability of reproducing the observed reversal asymmetry given the relatively large uncertainties in mean cluster direction. The outlined region encloses the 1σ confidence region. A broad range of field geometries are capable of producing this data set.

Table S1.

Source	Demag. type	Component range (mT)	Dec.	Inc.	N (fit)	Total steps	MAD	DANG	Dipolarity	Dec. (corrected)	Inc. (corrected)
**5c**	AF	12.5-150	349.5	10.1	13	27	3.8	2.2	0.61	356.0	10.1
**5d**	AF	2.5-240	141.9	35.8	16	34	16.2	8.4	0.71	148.4	35.4
**5h**	AF	10-52	339.2	-7.3	4	8	1.3	11.3	0.63	345.8	-8.3
**10b**	AF	2.5-110	181.9	15.9	7	21	19.8	16.8	0.79	185.8	17.6
**10e**	AF	0-110	327.9	36.8	8	22	19.3	14.4	0.60	334.6	44.2
**10m**	AF	48-210	359.8	32.5	8	19	17.7	11.9	0.60	3.7	36.6
**10p**	AF	0-110	161.6	-15.9	10	19	14.9	14.4	0.60	168.0	-19.8
		**Component range (°C)**									
**9a***	Thermal	0-275	194.2	37.8	6	10	16.1	N/A	0.64	185.5	45.5
**9a**	Thermal	275-325	7.2	-41.1	3	4	3.4	6.5	0.64	1.9	-47.8
**9b**	Thermal	255-340	226.5	34.4	7	7	17.3	1.9	0.62	220.0	53.5
**9f***	Thermal	80-280	96.9	27.4	4	9	11.3	N/A	0.64	93.0	27.4
**9f**	Thermal	280-340	9.5	-22.2	3	6	15.3	12.2	0.69	12.0	-24.5

Fit details for selected NRM demagnetization components. This includes all chromite-sulfide assemblages for which robust components were identified based on the selection criteria discussed in the Main Text. *N* represents the number of points used in the fit; because some demagnetization sequences were averaged before fitting, the total number of included demagnetization steps is larger for most components. The reported component ranges represent the full extent of unaveraged data included in the fitted component and may differ from the apparent fit range of the averaged data. The dipolarity parameter represents the average fraction of the root-mean-square (RMS) of the magnetic field map that can be represented by the best-fit dipole (*81*). Anisotropy corrections (final two columns) are discussed further in Supplementary Materials Section 3. Sources 5c, 5d, 9a, 9b are corrected with individual anisotropy tensors, and other sources are corrected with an average anisotropy tensor. Maximum angular deviation (MAD) quantifies the uncertainty in fit direction and the deviation angle (DANG) is the difference between the fitted component direction and the fit direction for the same points when forced through the origin. Nearly all reported components satisfy MAD > DANG, indicating they are within error of origin-trending and can be considered primary. The only exceptions to this are 5h and 9a, where MAD values are likely underestimated due to the small number of points used in fitting. * = low temperature component

Table S2.

Source	Mineral	Cr wt%	Fe wt%	O wt%	Ir wt%	Pd wt%	Pt wt%	Ru wt%	Totals
	CHR	30.17 ± 0.14	27.19 ± 0.17	28.29 ± 0.11					94.45
5d	CHR	31.56 ± 0.14	27.26 ± 0.17	29.54 ± 0.11					97.32
5d	CHR	33.05 ± 0.14	26.94 ± 0.17	30.21 ± 0.11					98.65
5b	CHR	32.82 ± 0.14	29.58 ± 0.17	32.08 ± 0.12					104.06
5b	CHR	34.33 ± 0.14	30.80 ± 0.18	33.90 ± 0.12					109.23
5b	CHR	34.58 ± 0.14	30.85 ± 0.18	32.28 ± 0.12					107.65
5b	CHR	35.17 ± 0.15	32.63 ± 0.18	35.63 ± 0.12					114.54
10i	CHR	32.94 ± 0.08	27.71 ± 0.09	30.55 ± 0.10	-0.02 ± 0.15	0.05 ± 0.04	-0.11 ± 0.07	0.03 ± 0.04	100.7
10d	CHR	31.74 ± 0.08	28.21 ± 0.09	30.21 ± 0.10	0.00 ± 0.15	0.00 ± 0.04	0.00 ± 0.07	0.00 ± 0.04	100.1
	OPX	1.15 ± 0.05	14.60 ± 0.13	52.14 ± 0.16					118.2
	OPX	0.65 ± 0.04	15.72 ± 0.14	52.81 ± 0.16					120.51

Semi-quantitative EDS abundances of key elements in non-sulfide minerals. Uncertainties reflect uncertainty in the calculated composition from the EDS data.

Table S3.

Source	Mineral	Fe wt%	S wt%	Ir wt%	Pd wt%	Pt wt%	Ru wt%	Totals
	PO	54.15 ± 0.22	27.09 ± 0.09	0.38 ± 0.11	0.00 ± 0.07	0.00 ± 0.12	0.00 ± 0.10	104.93
	PY	43.62 ± 0.20	47.39 ± 0.11	0.57 ± 0.10	0.00 ± 0.07	0.70 ± 0.12	0.57 ± 0.12	105.03
5d	PY	42.68 ± 0.20	48.25 ± 0.11	0.48 ± 0.10	0.00 ± 0.07	0.64 ± 0.12	0.00 ± 0.13	104.19
5d		42.54 ± 0.20	41.30 ± 0.10	0.76 ± 0.17	0.00 ± 0.07	0.50 ± 0.12	0.39 ± 0.12	103.62
5d		43.65 ± 0.20	41.03 ± 0.10	0.41 ± 0.10	0.00 ± 0.07	0.56 ± 0.12	0.44 ± 0.12	107.57
5b	PO	60.51 ± 0.23	40.35 ± 0.11	0.00 ± 0.14	0.00 ± 0.07	0.46 ± 0.13	0.38 ± 0.12	104.53
5b	PO	58.29 ± 0.23	37.13 ± 0.10	0.00 ± 0.14	0.00 ± 0.07	0.00 ± 0.13	0.00 ± 0.12	107.91
5b	PO	60.18 ± 0.23	38.93 ± 0.10	0.00 ± 0.14	0.00 ± 0.07	0.48 ± 0.13	0.46 ± 0.12	109.42
5b	PO	59.75 ± 0.23	37.41 ± 0.10	0.00 ± 0.14	0.00 ± 0.07	0.40 ± 0.13	0.37 ± 0.12	107.89
5b	PY	48.13 ± 0.21	57.29 ± 0.12	0.00 ± 0.14	0.00 ± 0.07	0.82 ± 0.13	0.70 ± 0.14	109.38
5b	PY	48.70 ± 0.21	57.41 ± 0.12	0.00 ± 0.14	0.00 ± 0.07	0.61 ± 0.13	0.74 ± 0.14	114.88
5b	PY	48.20 ± 0.21	55.73 ± 0.12	0.55 ± 0.11	0.00 ± 0.07	0.71 ± 0.12	0.84 ± 0.14	115.35
5b	PY	47.63 ± 0.21	52.70 ± 0.12	0.00 ± 0.14	0.23 ± 0.07	0.53 ± 0.13	0.52 ± 0.13	113.05
5b	PY	48.79 ± 0.21	57.39 ± 0.12	0.00 ± 0.14	0.00 ± 0.07	0.58 ± 0.13	0.79 ± 0.14	115.05
5b	PO	63.11 ± 0.24	47.34 ± 0.11	0.00 ± 0.15	0.00 ± 0.08	0.58 ± 0.13	0.59 ± 0.13	118.96
5b		53.05 ± 0.22	53.41 ± 0.12	0.00 ± 0.14	0.00 ± 0.07	0.58 ± 0.13	0.56 ± 0.13	117.3
5b	PO	63.88 ± 0.24	46.90 ± 0.11	0.58 ± 0.12	0.00 ± 0.08	0.52 ± 0.13	0.52 ± 0.13	125.59
10i		44.61 ± 0.11	36.23 ± 0.07	0.02 ± 0.16	0.05 ± 0.04	0.52 ± 0.08	0.04 ± 0.08	105.19
10i	PY	44.07 ± 0.11	53.82 ± 0.08	0.01 ± 0.16	0.14 ± 0.04	0.77 ± 0.08	0.21 ± 0.09	108
10i	PY	44.38 ± 0.11	54.56 ± 0.08	-0.18 ± 0.16	0.11 ± 0.04	0.78 ± 0.08	0.26 ± 0.09	108.63
10i	PY	44.06 ± 0.11	53.08 ± 0.08	0.01 ± 0.16	-0.02 ± 0.04	0.77 ± 0.08	0.14 ± 0.09	108.65
10i	PO	55.53 ± 0.12	39.64 ± 0.07	-0.08 ± 0.16	0.08 ± 0.04	0.58 ± 0.08	0.03 ± 0.08	105.84
10i	PO	58.59 ± 0.13	35.91 ± 0.07	-0.28 ± 0.16	0.08 ± 0.04	0.52 ± 0.09	0.11 ± 0.08	106.53
10i	PO	38.54 ± 0.11	25.55 ± 0.06	-0.14 ± 0.15	0.01 ± 0.04	0.40 ± 0.08	0.10 ± 0.07	99.15
10i	PO	40.78 ± 0.11	29.06 ± 0.06	-0.12 ± 0.16	0.12 ± 0.04	0.41 ± 0.08	0.24 ± 0.07	105.7
10d		45.47 ± 0.11	45.06 ± 0.07	-0.08 ± 0.16	0.07 ± 0.04	0.74 ± 0.09	0.31 ± 0.08	107.49
10d	PO	52.23 ± 0.12	38.19 ± 0.07	-0.03 ± 0.16	0.06 ± 0.04	0.60 ± 0.08	0.10 ± 0.08	101.1
10d	PY	45.72 ± 0.11	52.80 ± 0.08	-0.20 ± 0.16	-0.03 ± 0.05	0.74 ± 0.08	0.38 ± 0.09	107.63
10d		41.79 ± 0.11	40.00 ± 0.07	0.00 ± 0.16	0.00 ± 0.04	0.60 ± 0.08	0.30 ± 0.08	110.56
10d	PO	45.56 ± 0.11	34.75 ± 0.07	0.00 ± 0.16	0.00 ± 0.04	0.54 ± 0.08	0.00 ± 0.07	106.22
10d	PO	40.18 ± 0.11	25.42 ± 0.06	0.00 ± 0.15	0.00 ± 0.04	0.37 ± 0.08	0.00 ± 0.07	104.59
10d		50.50 ± 0.12	45.01 ± 0.07	0.00 ± 0.16	0.00 ± 0.05	0.63 ± 0.09	0.00 ± 0.09	113.94
10d	PO	55.68 ± 0.12	38.49 ± 0.07	0.00 ± 0.16	0.00 ± 0.04	0.54 ± 0.08	0.00 ± 0.08	104.99
10d		41.46 ± 0.11	37.11 ± 0.07	0.00 ± 0.15	0.00 ± 0.04	0.68 ± 0.09	0.00 ± 0.08	104.93
10d	PY	44.40 ± 0.11	50.62 ± 0.08	0.00 ± 0.16	0.00 ± 0.04	0.75 ± 0.08	0.36 ± 0.09	109.45
10d		42.18 ± 0.11	34.85 ± 0.07	0.00 ± 0.15	0.00 ± 0.04	0.58 ± 0.09	0.00 ± 0.07	114.55
10d	PY	45.21 ± 0.11	51.92 ± 0.08	0.00 ± 0.16	0.00 ± 0.05	0.78 ± 0.09	0.30 ± 0.09	111.09
10d	PY	44.97 ± 0.11	46.99 ± 0.07	0.00 ± 0.16	0.00 ± 0.04	0.70 ± 0.08	0.37 ± 0.09	111.01
10d	PY	47.10 ± 0.12	50.53 ± 0.08	0.00 ± 0.16	0.00 ± 0.05	0.78 ± 0.08	0.40 ± 0.09	108.29
10d	PY	43.72 ± 0.11	44.53 ± 0.07	0.00 ± 0.16	0.00 ± 0.04	0.67 ± 0.08	0.27 ± 0.08	107.85
10d	PO	53.92 ± 0.12	40.28 ± 0.07	0.00 ± 0.16	0.00 ± 0.04	0.57 ± 0.08	0.00 ± 0.08	106.27
10d	PY	45.38 ± 0.11	49.20 ± 0.08	0.00 ± 0.16	0.00 ± 0.04	0.77 ± 0.08	0.27 ± 0.09	109.95
10d		43.10 ± 0.11	41.56 ± 0.07	0.00 ± 0.15	0.00 ± 0.04	0.66 ± 0.08	0.00 ± 0.08	100.03
10d	PY	44.28 ± 0.11	50.71 ± 0.08	0.00 ± 0.16	0.00 ± 0.04	0.81 ± 0.08	0.00 ± 0.09	106.65
10d	PY	41.36 ± 0.11	41.97 ± 0.07	0.00 ± 0.16	0.00 ± 0.04	0.70 ± 0.08	0.39 ± 0.08	104.05

Semi-quantitative EDS abundances of key elements in sulfide minerals. Uncertainties reflect uncertainty in the calculated composition from the EDS data.

Table S4.

Source	Paleointensity type	Fit range (mT)	ΔNRM/ΔARM	Anisotropy correction	Paleointensity (µT)
**5c**	ARM	15-150	0.95 ± 0.08	0.94	60 ± 39
**5d**	ARM	27-240	0.15 ± 0.03	0.77	8 ± 5
**10p**	ARM	0-130	0.056 ± 0.029	0.97	4 ± 3
**10e**	ARM	27-100	0.69 ± 0.26	1.0	47 ± 35
**10b**	ARM	27-100	0.50 ± 0.15	1.0	33 ± 23
		**Fit range (°C)**	**-ΔNRM/ΔpTRM**		
**9f**	pTRM	275-340	0.10 ± 0.02	1.0	20 ± 4
**Source**	**Paleointensity type**	**ARM limit (µT)**	**NRM/ARM**	**NRM dipolarity**	**Paleointensity (µT)**
**5a**	Weak source	15	0.8	0.56	≤ 5.0 ± 3.7
**5b**	Weak source	20	0.5	0.10	≤ 6.7 ± 4.5
**5i**	Weak source	10	0.6	0.55	≤ 3.4 ± 2.8
**10i**	Weak source	20	0.7	0.61	≤ 6.7 ± 4.5

Single source paleointensity estimates. (Top) The type of paleointensity experiment performed, the range of demagnetization steps used to calculate the best-fit slope, the best-fit slope, the anisotropy correction factor, and the final computed source paleointensity estimate. The process of paleointensity estimation and calibration for these sources is discussed further in the Main Text and Section S5.1. (Bottom) Paleointensity limit data for identified weakly magnetized sources, including the ARM recording limit, the ratio of the NRM moment to the ARM moment at that bias field, the fit dipolarity, and the final paleointensity bound. Analysis of weakly magnetic sources, including the implications of the quantities reported here, is discussed further in Section S5.2.

Table S5.

Reproduced from Weiss & Tikoo (92)			
a	**Sample description**	**Domain state**	***f'***
1523	Synthetic monoclinic pyrrhotite	SD/PSD	1.9
1843	Synthetic monoclinic pyrrhotite	SD/PSD	2.3
1961	Synthetic monoclinic pyrrhotite	SD/PSD	2.4
2342	Synthetic monoclinic pyrrhotite	SD/PSD	2.9
2418	Synthetic monoclinic pyrrhotite	SD/PSD	3.0
2710	Synthetic monoclinic pyrrhotite	SD/PSD	3.3
2919	Synthetic monoclinic pyrrhotite	SD/PSD	3.6
3394	Synthetic monoclinic pyrrhotite	SD/PSD	4.1
3883	Terrestrial metasediment	MD?	4.7
4610	Terrestrial metasediment	MD?	5.6
4952	Terrestrial metasediment	MD?	6.0
4870	Terrestrial metasediment	MD?	5.9
5278	Terrestrial metasediment	MD?	6.4
2819	Terrestrial metasediment	MD?	3.4
808	Terrestrial metasediment	MD?	1.0

Estimated calibration coefficients for ARM paleointensity method (*f'*) used in this study. Values of the sIRM paleointensity method calibration coefficient (*a*) for different pyrrhotite samples as well as their assumed domain states are reproduced here from the table compiled by Weiss & Tikoo (*87*). To estimate uncertainties for our computed paleointensities, we estimated *f'* for pyrrhotite from these *a* values and the *f'/a* ratios computed for other minerals. This process is discussed in more detail in Section S5.1.

Table S6.

**Event**	**D#**	**Signatures**	**Temperature constraints**	**Age**
Igneous crystallization				4.091 ± 0.031 Ga (*18*, *21*)
Metamorphism/ possible impact	D1	Granular bands, homogenized compositions	875 ˚C (equilibrium)	4.1-4.0 Ga (*17*, *25*, *37*, *98, 99*)
Carbonate formation			18 ± 4 ˚C	3.95 ± 0.02 Ga (*29*, *30*)
~3.9 Ga impact	D2	$^{40}Ar/^{39}Ar$ plateau, siderite decomposition in carbonates, plagioclase glass formation, closed void spaces	≤ 320 ˚C (equilibrium)	~3.9 Ga (*36*, *99*, *101*)
~1.2 Ga impact	D3	$^{40}Ar/^{39}Ar$ plateau in some samples	≤ 325 ˚C (equilibrium)	1.16 ± 0.11 Ga (*37*) - 1.39 Ga (*101*)
Ejection from Mars	D4		≤ 320 ˚C (equilibrium)	15 Ma (*107*)
Arrival on Earth			≤ 40 ˚C	13,000 years ago (*103*)

Summary of major events in the history of ALH 84001.

REFERENCES AND NOTES

1. M. H. Acuña, J. E. P. Connerney, N. F, Ness, R. P. Lin, D. Mitchell, C. W. Carlson, J. McFadden, K. A. Anderson, H. Rème, C. Mazelle, D. Vignes, P. Wasilewski, P. Cloutier, Global distribution of crustal magnetization discovered by the mars global surveyor MAG/ER experiment. *Science* **284**, 790–793 (1999).

2. R. J. Lillis, S. Robbins, M. Manga, J. S. Halekas, H. V. Frey, Time history of the Martian dynamo from crater magnetic field analysis. *J. Geophys. Res. Planets* **118**, 1488–1511 (2013).

3. R. J. Lillis, H. V. Frey, M. Manga, Rapid decrease in Martian crustal magnetization in the Noachian era: Implications for the dynamo and climate of early Mars. *Geophys. Res. Lett.* **35**, 10.1029/2008GL034338 (2008).

4. S. J. Robbins, B. M. Hynek, R. J. Lillis, W. F. Bottke, Large impact crater histories of Mars: The effect of different model crater age techniques. *Icarus* **225**, 173–184 (2013).

5. C. Milbury, G. Schubert, C. A. Raymond, S. E. Smrekar, B. Langlais, The history of Mars' dynamo as revealed by modeling magnetic anomalies near Tyrrhenus Mons and Syrtis Major. *J. Geophys.Res.* **117**, 10.1029/2012JE004099 (2012).

6. L. L. Hood, K. P. Harrison, B. Langlais, R. J. Lillis, F. Poulet, D. A. Williams, Magnetic anomalies near Apollinaris Patera and the Medusae Fossae Formation in Lucus Planum, Mars, *Icarus* **208**, 118–131 (2010).

7. A. Mittelholz, C. L. Johnson, J. M. Feinberg, B. Langlais, R. J. Phillips, Timing of the martian dynamo: New constraints for a core field 4.5 and 3.7 Ga ago *Sci. Adv.* **6**, eaba0513 (2020).

8. S. Sakai, K. Seki, N. Terada, H. Shinagawa, T. Tanaka, Y. Ebihara, Effects of a weak intrinsic magnetic field on atmospheric escape from Mars. *Geophys. Res. Lett.* **45**, 9336–9343 (2018).

9. H. Egan, R. Jarvinen, Y. Ma, D. Brain, Planetary magnetic field control of ion escape from weakly magnetized planets. *Mon. Notices Royal Astron. Soc.* **488**, 2108–2120 (2019).

10. J. Caggiano, C. S. Paty, A multi-fluid magnetohydrodynamic investigation of Earth's magnetosphere during geomagnetic pole reversals, AGU Fall Meeting 2021, New Orleans, LA, 13–17 December 2021.

11. D. J. Hemingway, P. E. Driscoll, History and future of the Martian dynamo and implications of a hypothetical solid inner core. *J. Geophys. Res. Planets* **126**, e2020JE006663 (2021).

12. C. Milbury, G. Schubert, Search for the global signature of the Martian dynamo. *J. Geophys. Res. Planets* **115**, 10.1029/2010JE003617 (2010).

13. D. Boutin, J. Arkani-Hamed, Pole wandering of Mars: Evidence from paleomagnetic poles. *Icarus* **181**, 13–25 (2006).

14. C. Kutzner, U. R. Christensen, From stable dipolar towards reversing numerical dynamos. *Phys. Earth Planet. Inter.* **131**, 29–45 (2002).

15. P. Olson, U. R. Christensen, Dipole moment scaling for convection-driven planetary dynamos. *Earth Planet. Sci. Lett.* **250**, 561–571 (2006).

16. P. Rochette, J.-P. Lorand, G. Fillion, V. Sautter, Pyrrhotite and the remanent magnetization of SNC meteorites: A changing perspective on Martian magnetism. *Earth Planet. Sci. Lett.* **190**, 1–12 (2001).

17. L. E. Nyquist, D. D. Bogard, C.-Y. Shih, A. Greshake, D. Stöffler, O. Eugster, Ages and geologic histories of martian meteorites, in *Chronology and Evolution of Mars*, R. Kallenbach, J. Geiss, W. K. Hartmann, Eds. (Springer Netherlands, 2001), *Space Sciences Series of ISSI*, pp. 105–164.

18. A. Bouvier, J. Blichert-Toft, F. Albarède, Martian meteorite chronology and the evolution of the interior of Mars. *Earth Planet. Sci. Lett.* **280**, 285–295 (2009).

19. J. Gattacceca, P. Rochette, Toward a robust normalized magnetic paleointensity method applied to meteorites. *Earth Planet. Sci. Lett.* **227**, 377–393 (2004).

20. M. W. R. Volk, R. R. Fu, A. Mittelholz, J. M. D. Day, Paleointensity and rock magnetism of martian nakhlite meteorite miller range 03346: Evidence for intense small-scale crustal magnetization on mars. *J. Geophys. Res. Planets* **126**, e2021JE006856 (2021).

21. T. J. Lapen, M. Righter, A. D. Brandon, V. Debaille, B. L. Beard, J. T. Shafer, A. H. Peslier, A younger age for ALH84001 and its geochemical link to shergottite sources in mars. *Science* **328**, 347–351 (2010).

22. B. P. Weiss, H. Vali, F. J. Baudenbacher, J. L. Kirschvink, S. T. Stewart, D. L. Shuster, Records of an ancient Martian magnetic field in ALH84001. *Earth Planet. Sci. Lett.* **201**, 449–463 (2002).

23. B. P. Weiss, J. L. Kirschvink, F. J. Baudenbacher, H. Vali, N. T. Peters, F. A. Macdonald, J. P. Wikswo, A low temperature transfer of ALH84001 from Mars to Earth. *Science* **290**, 791–795 (2000).

24. A. H. Treiman, The history of Allan Hills 84001 revised: Multiple shock events. *Meteorit. Planet. Sci.* **33**, 753–764 (1998).

25. R. D. Ash, S. F. Knott, G. Turner, A 4-Gyr shock age for a martian meteorite and implications for the cratering history of Mars. *Nature* **380**, 57–59 (1996).

26. K. Terada, T. Monde, Y. Sano, Ion microprobe U-Th-Pb dating of phosphates in martian meteorite ALH 84001. *Meteorit. Planet. Sci.* **38**, 1697–1703 (2003).

27. B. P. Weiss, L. E. Fong, H. Vali, E. A. Lima, F. J. Baudenbacher, Paleointensity of the ancient Martian magnetic field. *Geophys. Res. Lett.* **35**, 10.1029/2008GL035585 (2008).

28. I. Halevy, W. W. Fischer, J. M. Eiler, Carbonates in the Martian meteorite Allan Hills 84001 formed at 18 ± 4 °C in a near-surface aqueous environment. *Proc. Natl. Acad. Sci. U.S.A.* **108**, 16895–16899 (2011).

29. L. E. Borg, J. N. Connelly, L. E. Nyquist, C.-Y. Shih, H. Wiesmann, Y. Reese, The age of the carbonates in martian meteorite ALH84001. *Science* **286**, 90–94 (1999).

30. B. L. Beard, J. M. Ludois, T. J. Lapen, C. M. Johnson, Pre-4.0 billion year weathering on Mars constrained by Rb–Sr geochronology on meteorite ALH84001. *Earth Planet. Sci. Lett.* **361**, 173–182 (2013).

31. R. P. Harvey, H. Y. M. Jr, A possible high-temperature origin for the carbonates in the martian meteorite ALH84001. *Nature* **382**, 49–51 (1996).

32. A. H. Treiman, H. E. F. Amundsen, D. F. Blake, T. Bunch, Hydrothermal origin for carbonate globules in Martian meteorite ALH84001: A terrestrial analogue from Spitsbergen (Norway). *Earth Planet. Sci. Lett.* **204**, 323–332 (2002).

33. K. L. Thomas-Keprta, S. J. Clemett, D. A. Bazylinski, J. L. Kirschvink, D. S. McKay, S. J. Wentworth, H. Vali, E. K. Gibson, M. F. McKay, C. S. Romanek, Truncated hexa-octahedral magnetite crystals in ALH84001: Presumptive biosignatures. *Proc. Natl. Acad. Sci. U.S.A.* **98**, 2164–2169 (2001).

34. A. J. Brearley, Magnetite in ALH 84001: An origin by shock-induced thermal decomposition of iron carbonate. *Meteorit. Planet. Sci.* **38**, 849–870 (2003).

35. A. H. Treiman, Submicron magnetite grains and carbon compounds in martian meteorite ALH84001: Inorganic, abiotic formation by shock and thermal metamorphism. *Astrobiology* **3**, 369–392 (2003).

36. G. Turner, S. F. Knott, R. D. Ash, J. D. Gilmour, Ar-Ar chronology of the Martian meteorite ALH84001: Evidence for the timing of the early bombardment of Mars. *Geochim. Cosmochim. Acta* **61**, 3835–3850 (1997).

37. W. S. Cassata, D. L. Shuster, P. R. Renne, B. P. Weiss, Evidence for shock heating and constraints on Martian surface temperatures revealed by $^{40}Ar/^{39}Ar$ thermochronometry of Martian meteorites. *Geochim. Cosmochim. Acta* **74**, 6900–6920 (2010).

38. A. H. Treiman, Uninhabitable and potentially habitable environments on Mars: Evidence from meteorite ALH 84001. *Astrobiology* **21**, 940–953 (2021).

39. K. L. Thomas-Keprta, S. J. Clemett, D. S. McKay, E. K. Gibson, S. J. Wentworth, Origins of magnetite nanocrystals in Martian meteorite ALH84001. *Geochim. Cosmochim. Acta* **73**, 6631–6677 (2009).

40. J. Fritz, A. Greshake, D. Stöffler, Micro-Raman spectroscopy of plagioclase and maskelynite in Martian meteorites: Evidence of progressive shock metamorphism. *Antarctic Meteorite Res.* **18**, 96–116 (2005).

41. T. L. North, G. S. Collins, T. M. Davison, A. R. Muxworthy, S. C. Steele, R. R. Fu, The heterogeneous response of martian meteorite Allan Hills 84001 to planar shock. *Icarus* **390**, 115322 (2023).

42. J. L. Kirschvink, A. T. Maine, H. Vali, Paleomagnetic evidence of a low-temperature origin of carbonate in the Martian meteorite ALH84001. *Science* **275**, 1629–1633 (1997).

43. M. Antretter, M. Fuller, E. Scott, M. Jackson, B. Moskowitz, P. Solheid, Paleomagnetic record of Martian meteorite ALH84001. *J. Geophys. Res.* **108**, 10.1029/2002JE001979 (2003).

44. J. P. Greenwood, H. Y. McSween Jr., Petrogenesis of Allan Hills 84001: Constraints from impact-melted feldspathic and silica glasses. *Meteorit. Planet. Sci.* **36**, 43–61 (2001).

45. J. L. Kirschvink, The least-squares line and plane and the analysis of palaeomagnetic data. *Geophys. J. Int.* **62**, 699–718 (1980).

46. T. Berndt, A. R. Muxworthy, K. Fabian, Does size matter? Statistical limits of paleomagnetic field reconstruction from small rock specimens. *J. Geophys. Res. Solid Earth* **121**, 15–26 (2016).

47. G. A. Paterson, D. Heslop, Y. Pan, The pseudo-Thellier palaeointensity method: New calibration and uncertainty estimates. *Geophys. J. Int.* **207**, 1596–1608 (2016).

48. P. L. McFadden, M. W. McElhinny, Classification of the reversal test in palaeomagnetism. *Geophys. J. Int.* **103**, 725–729 (1990).

49. L. Tauxe, *Essentials of Paleomagnetism* (University of California Press, 2010); www.degruyter.com/document/doi/10.1525/9780520946378/html

50. G. S. Watson, A test for randomness of directions. *Geophys. Suppl. Mon. Notices Royal Astron. Soc.* **7**, 160–161 (1956).

51. M. Jackson, Anisotropy of magnetic remanence: A brief review of mineralogical sources, physical origins, and geological applications, and comparison with susceptibility anisotropy. *Pure Appl. Geophys.* **136**, 1–28 (1991).

52. V. L. S. Bhimasankaram, Partial magnetic self-reversal of pyrrhotite. *Nature* **202**, 478–480 (1964).

53. K. L. Louzada, S. T. Stewart, B. P. Weiss, J. Gattacceca, N. S. Bezaeva, Shock and static pressure demagnetization of pyrrhotite and implications for the Martian crust. *Earth Planet. Sci. Lett.* **290**, 90–101 (2010).

54. P. Rochette, G. Fillion, R. Ballou, F. Brunet, B. Ouladdiaf, L. Hood, High pressure magnetic transition in pyrrhotite and impact demagnetization on Mars. *Geophys. Res. Lett.* **30**, 10.1029/2003GL017359 (2003).

55. K. Fabian, Thermochemical remanence acquisition in single-domain particle ensembles: A case for possible overestimation of the geomagnetic paleointensity. *Geochem. Geophys. Geosyst.* **10**, 10.1029/2009GC002420 (2009).

56. D. A. Crawford, P. H. Schultz, Electromagnetic properties of impact-generated plasma, vapor and debris. *Int. J. Impact Eng.* **23**, 169–180 (1999).

57. R. D. Wordsworth, L. Kerber, R. T. Pierrehumbert, F. Forget, J. W. Head, Comparison of "warm and wet" and "cold and icy" scenarios for early Mars in a 3-D climate model. *J. Geophys. Res. Planets* **120**, 1201–1219 (2015).

58. L. Carporzen, B. P. Weiss, S. A. Gilder, A. Pommier, R. J. Hart, Lightning remagnetization of the Vredefort impact crater: No evidence for impact-generated magnetic fields. *J. Geophys. Res. Planets* **117**, 1007 (2012).

59. L. M. Fairchild, N. L. Swanson-Hysell, S. M. Tikoo, A matter of minutes: Breccia dike paleomagnetism provides evidence for rapid crater modification. *Geology* **44**, 723–726 (2016).

60. H. J. Melosh, *Impact Cratering: A Geologic Process* (Oxford University Press, Clarendon Press, 1989); https://ui.adsabs.harvard.edu/abs/1989icgp.book.....M/abstract.

61. M. H. L. Deenen, C. G. Langereis, D. J. J. van Hinsbergen, A. J. Biggin, Geomagnetic secular variation and the statistics of palaeomagnetic directions. *Geophys. J. Int.* **186**, 509–520 (2011).

62. K. F. Sprenke, L. L. Baker, A. F. Williams, Polar wander on Mars: Evidence in the geoid. *Icarus* **174**, 486–489 (2005).

63. S. Bouley, D. Baratoux, I. Matsuyama, F. Forget, A. Séjourné, M. Turbet, F. Costard, Late Tharsis formation and implications for early Mars. *Nature* **531**, 344–347 (2016).

64. R. I. Citron, M. Manga, D. J. Hemingway, Timing of oceans on Mars from shoreline deformation. *Nature* **555**, 643–646 (2018).

65. P. Thomas, M. Grott, A. Morschhauser, F. Vervelidou, Paleopole reconstruction of Martian magnetic field anomalies. *J. Geophys. Res. Planets* **123**, 1140–1155 (2018).

66. C. L. Johnson, A. Mittelholz, B. Langlais, C. T. Russell, V. Ansan, D. Banfield, P. J. Chi, M. O. Fillingim, F. Forget, H. F. Haviland, M. Golombek, S. Joy, P. Lognonné, X. Liu, C. Michaut, L. Pan, C. Quantin-Nataf, A. Spiga, S. Stanley, S. N. Thorne, M. A. Wieczorek, Y. Yu, S. E. Smrekar, W. B. Banerdt, Crustal and time-varying magnetic fields at the InSight landing site on Mars. *Nat. Geosci.* **13**, 199–204 (2020).

67. B. Langlais, E. Thébault, A. Houliez, M. E. Purucker, R. J. Lillis, A new model of the crustal magnetic field of mars using MGS and MAVEN. *J. Geophys. Res. Planets* **124**, 1542–1569 (2019).

68. A. Genevey, Y. Gallet, C. G. Constable, M. Korte, G. Hulot, ArcheoInt: An upgraded compilation of geomagnetic field intensity data for the past ten millennia and its application to the recovery of the past dipole moment. *Geochem. Geophys. Geosyst.* **9**, 10.1029/2007GC001881 (2008).

69. A. J. Biggin, R. K. Bono, D. G. Meduri, C. J. Sprain, C. J. Davies, R. Holme, P. V. Doubrovine, Quantitative estimates of average geomagnetic axial dipole dominance in deep geological time. *Nat. Commun.* **11**, 6100 (2020).

70. P. Rochette, Crustal magnetization of Mars controlled by lithology or cooling rate in a reversing dynamo? *Geophys. Res. Lett.* **33**, 10.1029/2005GL024280 (2006).

71. S. Steele, R. R. Fu, A. I. Ermakov, R. I. Citron, R. J. Lillis, Z. Levitt, Could weakly magnetized martian basins reflect cooling in a reversing dynamo field? (2022).

72. J.-P. Williams, F. Nimmo, Thermal evolution of the Martian core: Implications for an early dynamo. *Geology* **32**, 97–100 (2004).

73. N. Michel, O. Forni, Mars mantle convection: Influence of phase transitions with core cooling. *Planet. Space Sci.* **59**, 741–748 (2011).

74. A. Pommier, C. J. Davies, R. Zhang, A Joint experimental-modeling investigation of the effect of light elements on dynamos in small planets and moons. *J. Geophys. Res. Planets* **125**, e2020JE006492 (2020).

75. S. Greenwood, C. J. Davies, A. Pommier, Influence of thermal stratification on the structure and evolution of the martian core. *Geophys. Res. Lett.* **48**, 10.1029/2021GL095198 (2021).

76. S. C. Stähler, A. Khan, W. B. Banerdt, P. Lognonné, D. Giardini, S. Ceylan, M. Drilleau, A. C. Duran, R. F. Garcia, Q. Huang, D. Kim, V. Lekic, H. Samuel, M. Schimmel, N. Schmerr,

D. Sollberger, É. Stutzmann, Z. Xu, D. Antonangeli, C. Charalambous, P. M. Davis, J. C. E. Irving, T. Kawamura, M. Knapmeyer, R. Maguire, A. G. Marusiak, M. P. Panning, C. Perrin, A.-C. Plesa, A. Rivoldini, C. Schmelzbach, G. Zenhäusern, É. Beucler, J. Clinton, N. Dahmen, M. van Driel, T. Gudkova, A. Horleston, W. T. Pike, M. Plasman, S. E. Smrekar, Seismic detection of the martian core. *Science* **373**, 443–448 (2021).

77. B. M. Jakosky, M. Slipski, M. Benna, P. Mahaffy, M. Elrod, R. Yelle, S. Stone, N. Alsaeed, Mars' atmospheric history derived from upper-atmosphere measurements of $^{38}Ar/^{36}Ar$. *Science* **355**, 1408–1410 (2017).

78. R. D. Wordsworth, The climate of early mars. *Annu. Rev. Earth Planet. Sci.* **44**, 381–408 (2016).

79. K. J. Mathew, K. Marti, Early evolution of Martian volatiles: Nitrogen and noble gas components in ALH84001 and Chassigny. *J. Geophys. Res. Planets* **106**, 1401–1422 (2001).

80. H. Gunell, R. Maggiolo, H. Nilsson, G. S. Wieser, R. Slapak, J. Lindkvist, M. Hamrin, J. D. Keyser, Why an intrinsic magnetic field does not protect a planet against atmospheric escape. *Astron. Astrophys.* **614**, L3 (2018).

81. R. R. Fu, E. A. Lima, M. W. R. Volk, R. Trubko, High-sensitivity moment magnetometry with the quantum diamond microscope. *Geochem. Geophys. Geosyst.* **21**, e2020GC009147 (2020).

82. L. Carporzen, B. P. Weiss, L. T. Elkins-Tanton, D. L. Shuster, D. Ebel, J. Gattacceca, Magnetic evidence for a partially differentiated carbonaceous chondrite parent body. *Proc. Natl. Acad. Sci.* **108**, 6386–6389 (2011).

83. D. R. Glenn, R. R. Fu, P. Kehayias, D. L. Sage, E. A. Lima, B. P. Weiss, R. L. Walsworth, Micrometer-scale magnetic imaging of geological samples using a quantum diamond microscope. *Geochem. Geophys. Geosyst.* **18**, 3254–3267 (2017).

84. M. W. R. Volk, R. R. Fu, R. Trubko, P. Kehayias, D. R. Glenn, E. A. Lima, QDM*lab*: A MATLAB toolbox for analyzing quantum diamond microscope (QDM) magnetic field maps. *Comput. Geosci.* **167**, 105198 (2022).

85. K. Righter, H. Yang, G. Costin, R. T. Downs, Oxygen fugacity in the Martian mantle controlled by carbon: New constraints from the nakhlite MIL 03346. *Meteorit. Planet. Sci.* **43**, 1709–1723 (2008).

86. A. R. Prunier Jr., D. A. Hewitt, Calculation of temperature-oxygen fugacity tables for H_2-CO_2 gas mixtures at one atmosphere total pressure. *GSA Bull.* **92**, 1039–1068 (1981).

87. B. P. Weiss, S. M. Tikoo, The lunar dynamo. *Science* **346**, 10.1126/science.1246753 (2014).

88. D. J. Dunlop, Ö. Özdemir, *Rock Magnetism: Fundamentals and Frontiers* (Cambridge University Press, 2010), *Cambridge Studies in Magnetism*; www.cambridge.org/core/books/rock-magnetism/6AEA7721B4B1C32E719C106583314989

89. A. P. Roberts, Q. Liu, C. J. Rowan, L. Chang, C. Carvallo, J. Torrent, C.-S. Horng, Characterization of hematite (α-Fe_2O_3), goethite (α-FeOOH), greigite (Fe_3S_4), and pyrrhotite (Fe_7S_8) using first-order reversal curve diagrams. *J. Geophys. Res. Solid Earth* **111**, 10.1029/2006JB004715 (2006).

90. P. Sattari, J. M. Brenan, I. Horn, W. F. McDonough, Experimental constraints on the sulfide- and chromite-silicate melt partitioning behavior of rhenium and platinum-group elements. *Econ. Geol.* **97**, 385–398 (2002).

91. D. C. Sassani, E. L. Shock, Solubility and transport of platinum-group elements in supercritical fluids: Summary and estimates of thermodynamic properties for ruthenium, rhodium, palladium, and platinum solids, aqueous ions, and complexes to 1000°C and 5 kbar. *Geochim. Cosmochim. Acta* **62**, 2643–2671 (1998).

92. J. W. Smith, D. A. Holwell, I. McDonald, Precious and base metal geochemistry and mineralogy of the Grasvally Norite–Pyroxenite–Anorthosite (GNPA) member, northern

Bushveld Complex, South Africa: Implications for a multistage emplacement. *Miner. Deposita* **49**, 667–692 (2014).

93. A. Agarwal, A. Kontny, T. Kenkmann, M. H. Poelchau, Variation in magnetic fabrics at low shock pressure due to experimental impact cratering. *J. Geophys. Res. Solid Earth* **124**, 9095–9108 (2019).

94. A. R. Muxworthy, P. A. Bland, T. M. Davison, J. Moore, G. S. Collins, F. J. Ciesla, Evidence for an impact-induced magnetic fabric in Allende, and exogenous alternatives to the core dynamo theory for Allende magnetization. *Meteorit. Planet. Sci.* **52**, 2132–2146 (2017).

95. D. L. Shuster, B. P. Weiss, Martian surface paleotemperatures from thermochronology of meteorites. *Science* **309**, 594–600 (2005).

96. Y. Yu, D. J. Dunlop, Ö. Özdemir, Are ARM and TRM analogs? Thellier analysis of ARM and pseudo-Thellier analysis of TRM. *Earth Planet. Sci. Lett.* **205**, 325–336 (2003).

97. A. H. Treiman, A petrographic history of martian meteorite ALH84001: Two shocks and an ancient age. *Meteoritics* **30**, 294–302 (1995).

98. B. P. Weiss, D. L. Shuster, S. T. Stewart, Temperatures on Mars from $^{40}Ar/^{39}Ar$ thermochronology of ALH84001. *Earth Planet. Sci. Lett.* **201**, 465–472 (2002).

99. D. D. Bogard, D. H. Garrison, Argon-39-argon-40 “ages” and trapped argon in Martian shergottites, Chassigny, and Allan Hills 84001. *Meteorit. Planet. Sci.* **34**, 451–473 (1999).

100. D. J. Barber, E. R. D. Scott, Shock and thermal history of Martian meteorite Allan Hills 84001 from transmission electron microscopy. *Meteorit. Planet. Sci.* **41**, 643–662 (2006).

101. M. Wadhwa, G. W. Lugmair, The formation age of carbonates in ALH84001. *Meteorit. Planet. Sci. Suppl.* **31**, A145 (1996).

102. K. Min, P. W. Reiners, High-temperature Mars-to-Earth transfer of meteorite ALH84001. *Earth Planet. Sci. Lett.* **260**, 72–85 (2007).

103. A. J. T. Jull, C. Courtney, D. A. Jeffrey, J. W. Beck, Isotopic evidence for a terrestrial source of organic compounds found in martian meteorites Allan Hills 84001 and Elephant Moraine 79001. *Science* **279**, 366–369 (1998).

104. S. C. Steele, Replication Data for: Paleomagnetic evidence for a long-lived, potentially reversing martian dynamo at ~3.9 Ga; https://doi.org/10.7910/DVN/PMZ5Z4.

105. S. C. Werner, The early martian evolution—Constraints from basin formation ages. *Icarus* **195**, 45–60 (2008).

106. H. Frey, Ages of very large impact basins on Mars: Implications for the late heavy bombardment in the inner solar system. *Geophys. Res. Lett.* **35**, 10.1029/2008GL033515 (2008).

107. O. Eugster, A. Weigel, E. Polnau, Ejection times of Martian meteorites. *Geochim. Cosmochim. Acta* **61**, 2749–2757 (1997).

108. D. A. Williams, R. Greeley, S. C. Werner, G. Michael, D. A. Crown, G. Neukum, J. Raitala, Tyrrhena Patera: Geologic history derived from *Mars Express* high resolution stereo camera. *J. Geophys. Res. Planets* **113**, 10.1029/2008JE003104 (2008).
